# Learning to Value Girls: Balanced Infant Sex Ratios at Higher Parental Education in the United States, 1969–2018

**DOI:** 10.1215/00703370-9968420

**Published:** 2022-06-01

**Authors:** Emily Rauscher, Haoming Song

**Affiliations:** Department of Sociology, Brown University, Providence, RI, USA; Department of Sociology, Brown University, Providence, RI, USA

**Keywords:** Education, Sex ratios, Gender inequality, Race/ethnicity

## Abstract

Infant sex ratios that differ from the biological norm provide a measure of gender status inequality that is not susceptible to social desirability bias. Ratios may become less biased with educational expansion through reduced preference for male children. Alternatively, bias could increase with education through more access to sex-selective medical technologies. Using National Vital Statistics data on the population of live births in the United States for 1969–2018, we examine trends in infant sex ratios by parental race/ethnicity, education, and birth parity over five decades. We find son-biased infant sex ratios among Chinese and Asian Indian births that have persisted in recent years, and regressions suggest son-biased ratios among births to Filipino and Japanese mothers with less than a high school education. Infant sex ratios are more balanced at higher levels of maternal education, particularly when both parents are college educated. Results suggest greater equality of gender status with higher education in the United States.

## Introduction

Son-biased infant sex ratios document unequal access to life in the United States among children of Chinese, Asian Indian, and Korean parents (Abrevaya 2009; Almond and Edlund 2008; Almond and Sun 2017; Ost and Dziadula 2016). Efforts to reduce bias in birth ratios have focused on economic development, cultural or normative changes, and policies to limit access to sex-selective abortion (Chung and Das Gupta 2007; Rebouche 2015). In the United States and other developed countries, however, relatively little is known about factors related to changes in son-biased sex ratios (Mussino et al. 2018).

Education may be related to son-biased sex ratios in the United States through cultural change and through economic and medical resources. By shaping cultural preferences (Baker 2014), education may reduce preference for male children among expecting parents. For example, higher education could provide a greater sense of individual freedom from patriarchal cultures and encourage more cosmopolitan or egalitarian cultural attitudes (Baker 2014; Echávarri and Ezcurra 2010; Weitzman 2018), yielding less biased child sex ratios. Alternatively, by increasing income and medical knowledge, education could enable more access to sex-selective technologies such as prenatal blood tests and abortion, which could yield elevated male-biased child sex ratios (Almond and Edlund 2008; Almond et al. 2019; Almond and Sun 2017; Bhat and Zavier 2007; Hesketh and Xing 2006).

Education is associated with child sex ratios in Asian countries (Das Gupta 2017; Echávarri and Ezcurra 2010; Gietel-Basten et al. 2018; Pande and Astone 2007; Zaidi and Morgan 2016). Evidence suggests that shifts in culture and social norms play a larger role in changing son-biased sex ratios (accounting for nearly three fourths of the decline in South Korea) than development measures, including education and urbanization (Chung and Das Gupta 2007). However, educational expansion preceded the change in South Korea and could be a necessary precondition for cultural and normative changes. Furthermore, little is known about the relationship between education and son-biased ratios in the United States or how that relationship has changed over time. One exception provides mixed evidence, suggesting that the relationship varies by parental ethnicity (Abrevaya 2009), yet research has focused on relatively few ethnic groups.

Ultrasound technology made prenatal sex determination widely available in the United States beginning in the 1970s and 1980s (Abrevaya 2009). How have infant sex ratios by parental race and ethnicity changed since the 1970s? What is the relationship between education and infant sex ratios? How does that relationship differ by parental race/ethnicity?

We examine variation in infant sex ratios among U.S.-born children by birth order, parental education, and parental racial/ethnic identification using annual administrative birth data from the National Vital Statistics System. We find son-biased infant sex ratios—particularly at high birth orders—among Chinese and Asian Indian births that persist in recent years. Son-biased ratios were high among Korean births in previous decades but declined over time. Regression estimates suggest biased infant sex ratios among third births to Chinese, Asian Indian, Korean, Japanese, and Filipinos, but only among mothers with low education levels. We find more balanced infant sex ratios at higher levels of maternal education. These results hold when controlling for time-varying aggregate measures of economic and cultural characteristics by racial/ethnic category from U.S. Census and American Community Survey (ACS) data. Less male bias is also observed at higher levels of parental education when examining child sex ratios conditional on the sex of previous children in the ACS. Results suggest that educational expansion is related to more balanced sex ratios in the United States.

## Background

Substantial progress among women in education and employment over the past several decades (DiPrete and Buchmann 2013; England 2010; Jackson 2010) may lead policymakers and the public to believe that women and men have achieved equality in the United States. However, gender inequality measures often focus on resources, neglecting an equally powerful dimension: status, or widely shared cultural beliefs about esteem and honor (Ridgeway 2014). The “stalled gender revolution” (England 2010; England et al. 2020) suggests that progress toward gender equality in status may also have stalled since the mid-1990s.

Measuring gender equality of status is difficult given social desirability bias, which can increase with education (Jackman 1978). Those with higher education may be more attuned to the socially desirable answers regarding gender equality and so provide more biased answers to survey or interview questions about gender status (Jackman 1978; Jackman and Muha 1984). Documenting trends in gender status requires a consistent measure that is not subject to social desirability bias.

Furthermore, aggregate trends may hide heterogeneity by race/ethnicity. Gender status is complex, with variation among categorical social groups (Ridgeway 2014; Sidanius et al. 2004; Tilly 1998). For example, Asian Americans from traditionally patriarchal ethnic backgrounds (e.g., Chinese and Asian Indian) (Almond and Edlund 2008; Almond and Sun 2017) may hold strong male-biased preferences. Examining variation within and between ethnic groups provides more information about where progress toward gender equality in status has stalled. Our study identifies correlates of infant sex ratios and contributes to the broader debate about the stalled gender revolution. By documenting trends in a measure of gender status that is not susceptible to social desirability bias, we assess whether the stalled gender revolution holds for status.

### Infant Sex Ratios Measure Gender Equality of Status

Prior quantitative research measured gender status through surveys (Knight and Brinton 2017) or experiments (Correll et al. 2007; Quadlin 2018), yet both approaches have drawbacks. By directly asking gender attitude questions, survey measurement suffers from social desirability bias. Experimental measurement, owing to the nature of its design, frequently sacrifices lower external validity for higher internal validity and thus findings may not generalize to broader social contexts or other groups (Bertrand and Duflo 2016).

Biased sex ratios at birth provide an objective measure of gender inequality that is based on observed administrative data rather than self-reported data, is not subject to social desirability bias in reporting (Fisher 1993), and maintains high external validity. Biased child sex ratios differ from the biological norm of 1.05–1.06 males to females (Chahnazarian 1988; Renkonen et al. 1962). Male-biased sex ratios are significantly higher than the biological norm and female-biased ratios are significantly lower. Genetic differences do not explain variation in infant sex ratios, because there is no genetic contribution to individual offspring sex ratio (Zietsch et al. 2020). Male:female ratios higher than the biological norm indicate manipulation through sex-selective medical techniques (sex-selective abortion, in vitro fertilization, or sperm sorting; Abrevaya 2009; Almond and Edlund 2008; Chen et al. 2013), practices used by parents with unequal gender preferences. Thus, male-biased sex ratios indicate unequal gender status.

Male:female ratios lower than the biological norm are associated with exposure to environmental pollution and chronic socioeconomic stress (Figá-Talamanca and Petrelli 2000; Goldsmith et al. 1984; Grech 2017, 2018, 2019; Mocarelli et al. 2000; Sakamoto et al. 2001; Smith and Von Behren 2005; Whorton et al. 1994). African Americans and American Indians experience high exposure to pollution (Mohai et al. 2009; Zwickl et al. 2014). These groups also have the highest poverty rates in the United States (27% and 21%, respectively; Kaiser Family Foundation 2019) and experience discrimination in educational, labor market, health, and criminal justice settings (Findling et al. 2019; Greene et al. 2006; Noguera 2003; Pager 2003; Skiba et al. 2002). The combination of poverty and discrimination creates chronic socio-economic stress, which can result in lower male:female sex ratios (Grech 2017, 2018, 2019). As a result of these environmental exposures, African Americans and American Indians likely have low male:female ratios at birth.

Male-biased sex ratios indicate strong gender inequality because sex-selective practices have high physical, emotional, and financial costs, likely undertaken only by those with a strong preference for male children. Child sex ratios therefore do not capture less extreme gender inequality. Differences in prenatal care by infant sex provide an additional measure that includes less extreme gender inequality than birth ratio. Although some research has found little difference in prenatal care by fetal sex (Lhila and Simon 2008), recent evidence suggests the relationship may vary by both maternal education and ethnicity (Almond and Cheng 2021). For example, some work suggests unequal infant and child health by sex among births to Chinese and Asian Indian parents in the United States (Almond and Cheng 2021; Almond and Sun 2017; Muchomba and Chatterji 2020).

Unbalanced sex ratios shape multiple demographic and cultural aspects of group life, including inequality of gender status through romantic partnership opportunities and relationship stability (Guttentag and Secord 1983). Unequal prenatal care captures less extreme gender inequality, yet still has important implications given the effects of infant health on later life outcomes (including childhood mortality and adult education and earnings) (Black et al. 2007; Conley et al. 2003; Royer 2009). We focus primarily on infant sex ratios and examine whether inequality of prenatal care follows similar patterns.

Son-biased sex ratios over the biological norm are well documented in India, China, and South Korea (Bhat and Zavier 2007; Chung and Gupta 2007; Jha et al. 2006; Park and Cho 1995; Tuljapurkar et al. 1995; Zaidi and Morgan 2016; Zeng et al. 1993). Son preference has also been observed in rural areas or in older generations in the Philippines and Japan (Kureishi and Wakabayashi 2011; Stinner and Mader 1975), as well as among certain groups in Western countries, including Germany, Norway, Australia, and the United States (Abrevaya 2009; Almond and Edlund 2008; Carol and Hank 2020; Edvardsson et al. 2018; Lillehagen and Lyngstad 2018; Mussino et al. 2018).

The preference for sons emerges at higher birth orders (higher parity). Among U.S.-born children of Chinese, Asian Indian, and Korean parents, the male bias emerges at second and third births when there was no previous son (Almond and Edlund 2008; Almond and Sun 2017). Less is known about son bias among other U.S. racial/ethnic groups or how son preferences have changed over time. Two related studies in the United States examined relatively few racial/ethnic groups and ended in 2002 (Egan et al. 2011; Mathews and Hamilton 2005). Several changes, including reduced abortion access in many states, more egalitarian gender views (e.g., in South Korea) (Chung and Das Gupta 2007), and increased postsecondary education, could have altered infant sex ratios since 2002. We expect to find:

*Hypothesis 1a*: Male-biased sex ratios at higher birth orders among births to Chinese and Asian Indian mothers.

*Hypothesis 1b*: Female-biased sex ratios among births to African American and American Indian mothers.

### Education and Gender Inequality

Education plays important and complex roles in gender inequality (Mickelson 1989). Gender inequality manifests in school achievement, participation, course taking, and educational attainment, with implications in the family and labor market (DiPrete and Buchmann 2013; Dumais 2002; Legewie and DiPrete 2012; Sutton et al. 2016; Weitzman 2018). Schools also provide an important context for the development and performance of gender identity (Meadow 2018; Morris 2008; Pascoe 2011; Thorne 1993). Yet the educational implications of gender inequality may manifest before students enter school and even before birth. Specifically, education may shape parental gender preferences and influence gender inequality in the most important aspect of life chances: the likelihood of being born.

Preference for male children may vary by education. For example, education may be associated with less biased infant sex ratios by altering cultural views (Baker 2014), including preference for male children. Education can provide a sense of individual freedom from traditional cultures and increase egalitarian attitudes (Baker 2014; Echávarri and Ezcurra 2010). If education promotes gender egalitarian preferences, then higher education will be associated with infant sex ratios that are less male-biased. Therefore, we expect more balanced child sex ratios among Chinese and Asian Indian births at higher levels of parental education.

Education may also be associated with more balanced sex ratios among groups with elevated female births. Environmental exposure to pollution and socioeconomic stress are linked to female-biased sex ratios (e.g., Grech 2017; Sakamoto et al. 2001), and these exposures are lower at higher levels of education and income (Bell and Ebisu 2012; Samet and White 2004). For example, higher education can reduce exposure to pollution and chronic socioeconomic stress through higher income and knowledge. Therefore, we expect more balanced male:female ratios among African American and American Indian births at higher levels of parental education.

*Hypothesis 2a*: More balanced infant sex ratios at higher levels of maternal education.

Alternatively, education may provide more resources and access to sex-selective technologies (e.g., prenatal blood tests, abortion), which could yield elevated male-biased child sex ratios (Almond and Edlund 2008; Almond and Sun 2017; Bhat and Zavier 2007; Hesketh and Xing 2006). For example, following a reform that increased household income in China, Almond and colleagues (2019) found that male-biased sex ratios increased more among mothers with higher education. In addition to resources, education could also increase sex selection by reducing fertility, placing more importance on having a son at first or second parity and increasing male-biased ratios overall. Evidence from Canada suggests that sex-selective practices occur at earlier parity with lower fertility (Almond et al. 2013). Taken together, if education encourages lower fertility (Lesthaeghe 2010), then infant sex ratios could become more biased with increased education. If education facilitates sex-selective practices through higher resources or lower fertility, then higher education will be associated with more biased child sex ratios.

*Hypothesis 2b*: More male-biased infant sex ratios at higher levels of maternal education.

Research that explicitly examined variation by education found higher male-biased sex ratios among mothers with higher education in India (Bhat and Zavier 2007; Jha et al. 2006). In the United States, Abrevaya (2009) examined infant sex ratios through 2005 and found similar sex ratios by maternal education among Asian Indian mothers, but more male-biased ratios among Chinese mothers with higher education. Educational expansion and rising immigration from many Asian countries warrant examination of additional ethnic groups using more recent data. For example, from 2000 to 2019, the number of migrants has increased 163% from India, 88% from China, 49% from the Philippines, 40% from Vietnam, and 20% from Korea, and decreased by 4% from Japan (Migration Policy Institute 2020). Population composition changes call for examination of variation in infant sex ratios in recent years among more ethnic groups.

### Economic and Cultural Changes and Gender Equality

Changes in aggregate-level economic and cultural attributes could contribute to gender equality over time. Modernization theory generally predicts cultural changes (e.g., increased individualism and liberalism) from economic development, industrialization, and urbanization (Inglehart and Baker 2000; Inglehart et al. 2017). These cultural changes could occur rapidly within generations (e.g., views on same-sex marriage) or more slowly with compositional shifts across generations. Norms of individual choice have become more dominant in recent decades, and the speed of change has accelerated (Inglehart and Baker 2000). Modernization theory would expect son preference to decrease quickly over time along with the rise in individualism and liberalism.

Similar to modernization theory, the second demographic transition theory also predicts an equalizing trend: when societies place more value on self-expression and individualization, both fertility and son preference should decline (Lesthaeghe 2010).

Cultural norm diffusion, accompanied and accelerated by the development of mass media and progressive social policy, is another powerful mechanism linking education, economic advancement, and infant sex ratios (den Boer and Hudson 2017; Guilmoto 2009). This is perhaps most evident in the case of South Korea, where bias in child sex ratios began decreasing among the socially elite (tertiary-sector workers and the college educated) a few years before it did among lower status groups (Chung and Das Gupta 2007). Taken together, these theories generally predict more balanced infant sex ratios and declining fertility over time with modernization and educational expansion.

*Hypothesis 3*: More balanced infant sex ratios and declining fertility since 1969.

In contrast, critics challenge modernization theory’s unilinear and developmental assumptions and the lack of consideration of local culture and institutions (Zaidi and Morgan 2017). For instance, gender equality could vary nonlinearly across years with economic recessions, changes in migration rates, or other temporal changes, and accounting for these potential year-specific changes is required to assess trends. In addition, despite high levels of industrialization and urbanization, cultural persistence helps to maintain biased sex ratios among immigrant populations in developed societies (Almond et al. 2013; Mussino et al. 2018). For the same reason, infant sex ratios tend to persist within groups over time and examining within-group variation (e.g., over time and by education) provides information about when and how gender equality improves.

Education is correlated with both cultural and economic measures (e.g., family income). In immigrant societies, both economic and cultural characteristics (e.g., generational status or age at immigration, which serves as a proxy for cultural assimilation) may account for variation in gender preference within and between racial/ethnic groups (Bongaarts 2013; Guilmoto 2009). The extent to which economic and cultural characteristics account for variation in inequality of infant sex ratios remains an open question. Holding constant economic and cultural characteristics is important in identifying the relationship between education and infant sex ratios.

### Educational Homogamy and Infant Sex Ratios

Part of the persistence of infant sex ratios may reflect that decisions about whether to terminate a pregnancy occur at a local level: within couples. According to family systems theory, families operate as a unit, with complex emotional interactions and inter-dependencies (Fletcher 2009; Gihleb and Lifshitz 2016; Kerr 2000; Minuchin 1985). Interrelationships among family members and their characteristics have implications for the family and could also influence the sex selection of future children. Family systems theory suggests that—in addition to the absolute level of education—the relative education of parents should also have implications for the family. For example, beyond a mother’s absolute level of education, her education relative to the father’s also has implications for infant health (Rauscher 2020). However, the benefits of parental educational homogamy for infant health are limited to mothers with relatively high education (Rauscher 2020). Similarly, educational homogamy at high levels of maternal education may also be related to greater equality of child sex ratios.

Beyond the absolute levels of parental education, therefore, family systems theory suggests that the relationship between education and infant sex ratios will be amplified when parents have the same level of education. Parents with equal education may agree more on parenting practices and preferences (Beck and Gonzalez-Sancho 2009; Martin et al. 2007). For example, both parents having a college education could increase agreement on cultural preferences such as gender equality. In that case, an association between maternal college education and infant sex ratios should increase with paternal college education. The more egalitarian cultural views promoted by higher education (Baker 2014; Echávarri and Ezcurra 2010) should be amplified when both parents share those views. Similarly, when both parents have low levels of education, they may have more biased preferences.

*Hypothesis 4*: The relationship between infant sex ratios and maternal education is stronger among educationally homogamous parents.

## Methods

### Data

Using annual U.S. administrative birth records from the National Vital Statistics System (NVSS; NCHS 2018), we examine trends in aggregate infant sex ratios by maternal racial/ethnic category, birth order, and maternal education from 1969 to 2018. These microdata are based on administrative birth certificate records and provide the most complete and accurate information about births in the United States. We examine births at parities 1–3, with maternal education and race/ethnicity information, to mothers who are Chinese, Asian Indian, Korean, Filipino, Vietnamese, White, African American, or American Indian, yielding a total of 137,039,340 live births. Paternal education information is not available in years 1995–2010. Limiting analysis to births with both maternal and paternal education (to test Hypothesis 4) yields a total of 71,843,472 live births. Table A1 in the online appendix shows the proportion missing information for each measure in the NVSS data.

Because infant sex ratios may depend on economic and cultural characteristics (Bongaarts 2013; Guilmoto 2009), we use U.S. Census 1970–2000 and ACS 2001–2018 data from IPUMS (Ruggles et al. 2018) to control for aggregate economic and cultural characteristics by race/ethnicity group.

Previous research has found that child sex ratios are most elevated in cases when there are previous female children (Almond and Edlund 2008); NVSS data do not include information about the sex of previous children. In sensitivity analyses, we pool ACS data for years 2000–2018 to examine child sex ratios by parental race/ethnicity and education, conditional on the sex of previous children. These data rely on all children being present in the household and have smaller sample sizes than NVSS data, but allow a valuable sensitivity check (our Supplementary Online Appendix provides more details). Note that we examine infant sex ratios in NVSS data and child sex ratios in ACS data. Infant and child sex ratios examine gender inequality at different child ages.

### Measures

Annual infant sex ratios are calculated as the number of male infants divided by the number of female infants in each maternal racial/ethnic category and at each parity. Parity is the birth order of the infant, relative to previous live births. Maternal racial/ethnic category is based on self-report. Results are consistent when using paternal racial/ethnic category or parents with the same racial/ethnic identification. Previous research indicates male-biased child ratios within detailed ethnic categories, including Chinese, Asian Indian, and Korean. We examine those groups and add to the literature by examining trends in other groups as well, including Filipino, Vietnamese, White, African American, and American Indian. Ethnic categories are less detailed in the early years of the NVSS data; Hispanic/Latinx ethnicity is not available until 1978, and we examine variation by race regardless of Hispanic ethnicity for consistency across all years. Asian Indian, Korean, and Vietnamese ethnic categories become available in 1992.

We further disaggregate infant sex ratios by maternal education within race/ethnicity and parity. Education categories include those with less than high school, a high school degree, some college, or at least a college degree. We create separate indicators for whether the mother or father has any college education (some college or a bachelor’s degree). Indicators for parents with or without any college education capture exposure to the cosmopolitan culture and egalitarian norms of college and also the higher earning potential associated with college attendance (Averett and Burton 1996; Baker 2014; Giani et al. 2020; Greenwood 1975). To test Hypothesis 4, we calculate separate infant sex ratios by both maternal and paternal education within race/ethnicity and parity categories in years 1969–1994 and 2011–2018 (when paternal education is available).

Aggregate measures are calculated using nonmissing values. For example, the number of male and female births in a particular race/ethnicity category and birth order excludes birth records with missing information for maternal race/ethnicity, infant sex, and birth order. Missing rates (see Table A1 in the online appendix) are less than 1% on most key measures, including maternal race/ethnicity, infant sex, and birth order. Maternal education is missing for 11% of records. As states adopted new birth certificate formats after 1997 and 2008, maternal education is not included on a substantial subset of birth certificates in certain state-years. In these cases, maternal education is not selectively unreported by mothers, but is excluded from birth certificates in certain states and years for reasons unrelated to infant sex ratios. In addition, a substantial portion of birth records are missing paternal information: 15% are missing paternal race/ethnicity, 14% are missing paternal age, and 53% are missing paternal education when including all years. Paternal education was not included on any NVSS birth records in 1995–2010. When excluding those years, 26% of birth records are missing paternal education. Our main analyses use maternal race/ethnicity and education information because of high missing rates for paternal information. Analyses using paternal information could yield biased results if mothers who were more likely to practice infant sex selection were more or less likely to report paternal information.

Using census and ACS data, we calculate mean economic and cultural measures for each detailed race/ethnicity category from the years 1970, 1980, 1990, 2000, 2001–2005, 2006–2010, 2011–2015, and 2016–2018. These measures are calculated separately among men and women to capture potential gender differences within racial/ethnic groups. *Economic measures* include home ownership rate, mean home value, mean family income (all currency is inflation-adjusted to 2018 dollars), proportion not in the labor force, and proportion living on a farm. *Cultural measures* include proportions foreign-born, noncitizen, Hispanic, and living in a three-generation household (e.g., grandparents, parents, and children), and mean years in the United States among those born outside the country. The proportion of women not in the labor force is included in the economic measures; we also include it in the cultural measures because it provides a proxy for traditional gender roles. Annual infant sex ratio data are linked to aggregate census or ACS measures from the nearest available year. For example, birth years 1969–1974 are linked to aggregate values from the 1970 census, and birth years 1975–1984 are linked to aggregate values from the 1980 census. Table A2 in the online appendix identifies the data source used to calculate aggregate values for each year of infant sex ratios.

### Analyses

We predict annual aggregate infant sex ratios by maternal racial/ethnic category, education, and birth order in ordinary least-squares regression models that include indicators for each racial/ethnic category, birth order, and year. Bootstrapped standard errors are calculated using 100 repetitions and are stratified by race/ethnicity because infant sex ratios tend to be similar within groups across years (Angrist and Pischke 2009). Sensitivity analyses using a higher or lower number of repetitions and without stratifying by race/ethnicity yield consistent results.

(1) $${\text{Ratio}}_{ijkt}=\beta_{i}{\text{Race/Ethnic}}_{i}+\beta_{j}{\text{Birth}\;\text{Order}}_{j}+{\;\text{Year}}_{t}+W_{ijkt}+X_{it}+Z_{ikt}+\varepsilon_{ijkt}.$$

Equation (1) predicts the male:female infant sex ratio in maternal race/ethnic category (*i*), birth order (*j*), education category (*k*), and year (*t*). Coefficients for race/ethnicity categories (β_*i*_) test whether infant sex ratios differ by race/ethnicity across all years (Hypothesis 1). To test whether infant sex ratios differ significantly by maternal education (Hypothesis 2), ratios are predicted with coefficients for maternal education categories (β_*k*_) interacted with each race/ethnicity category. Coefficients for a continuous year measure interacted with race/ethnicity categories test whether trends in infant sex ratios differ by race/ethnicity (Hypothesis 3). We test Hypothesis 4 using aggregate infant sex ratios further disaggregated by paternal education category. We stratify the sample by paternal college education and repeat analyses for Hypothesis 2, with interaction terms for maternal education and race/ethnic category. We test whether coefficients for maternal college education by race/ethnicity differ significantly in the models limited to fathers with and without a college education (Clogg et al. 1995; Paternoster et al. 1998). We calculate

$$z=\left(\beta_{Coll}-\beta_{NoColl}\right)/\sqrt{SE_{Coll}^{2}+SE_{NoColl}^{2}},$$

where β_*Coll*_ indicates the coefficient for the interaction between maternal college and Chinese ethnicity (for example) in the model limited to fathers with college education, and β_*NoColl*_ is the same coefficient in the model limited to fathers with no college (Paternoster et al. 1998). These *z* tests indicate whether the relationship between maternal education and infant sex ratio is stronger if parents are educationally homogamous (Hypothesis 4).

We fit all models with and without controls for (1) parental characteristics (*W*_*ijkt*_), including maternal and paternal age, marital status, and whether the mother is a U.S. resident (parental characteristics are measured at the same unit of analysis as infant sex ratios, i.e., race/ethnicity–birth order–education–year category); (2) time-varying measures (*X_it_*) of mean economic and cultural characteristics measured for each racial/ethnic category from census and ACS data; and (3) time-varying fertility measures (proportion of births at first, second, and third parity) by racial/ethnic and education category (*Z_ikt_*). We fit models with and without these controls because previous work has suggested that infant sex ratios may depend on fertility and on these economic and cultural characteristics (Almond et al. 2013; Bongaarts 2013; Guilmoto 2009). An association between parental education and infant sex ratios could be confounded by fertility, economic, or cultural measures without holding them constant. Our aggregate economic and cultural characteristics measures are coarse and include substantial error.

### Sensitivity Analyses

In addition to infant sex ratios, fertility may also vary by education (Almond et al. 2013; Lesthaeghe 2010). We use the same methods (excluding fertility controls in the final model) to examine variation in mean live birth order by maternal race/ethnicity, education, and year. Live birth order excludes fetal deaths and is the number of live births the mother has had, including the current birth. Mean live birth order provides a proxy for fertility. Comparing results for fertility and sex ratios allows us to assess whether sex selection increased as fertility declined within ethnic groups (Almond et al. 2013).

We repeat the main analyses predicting inequality of prenatal care as a less extreme measure of gender inequality than unbalanced infant sex ratios. For comparability with infant sex ratios, we measure inequality of prenatal care as the male:female ratio of prenatal visits. Specifically, within each race/ethnicity, birth order, education, and year category, we calculate the ratio of the mean number of prenatal visits for male births divided by the mean number of prenatal visits for female births. As with infant sex ratios, higher values indicate male bias (more prenatal care for male fetuses).

We use maternal racial/ethnic identification in primary analyses to avoid potential sample selection bias due to unequal likelihood of missing paternal information by child sex. For example, when having male children, married parents are less likely to divorce and unmarried mothers are more likely to marry the child’s father (Dahl and Moretti 2008; Lundberg and Rose 2003). Therefore, female infant birth records may be more likely to have missing paternal information, which could bias estimates. Sensitivity analyses using paternal racial/ethnic identification or limiting analyses to births to parents in the same race/ethnicity category yield qualitatively similar results.

Sensitivity analyses examine child sex ratios at third parity in ACS data for 2000–2018 by parental race/ethnicity and education, conditional on the sex of the two previous children. Using individual-level data for each third child, we predict the likelihood that the child is a boy when the two previous children are girls compared to two previous boys. The Supplementary Online Appendix provides additional details.

## Results

**Fig. 1:**
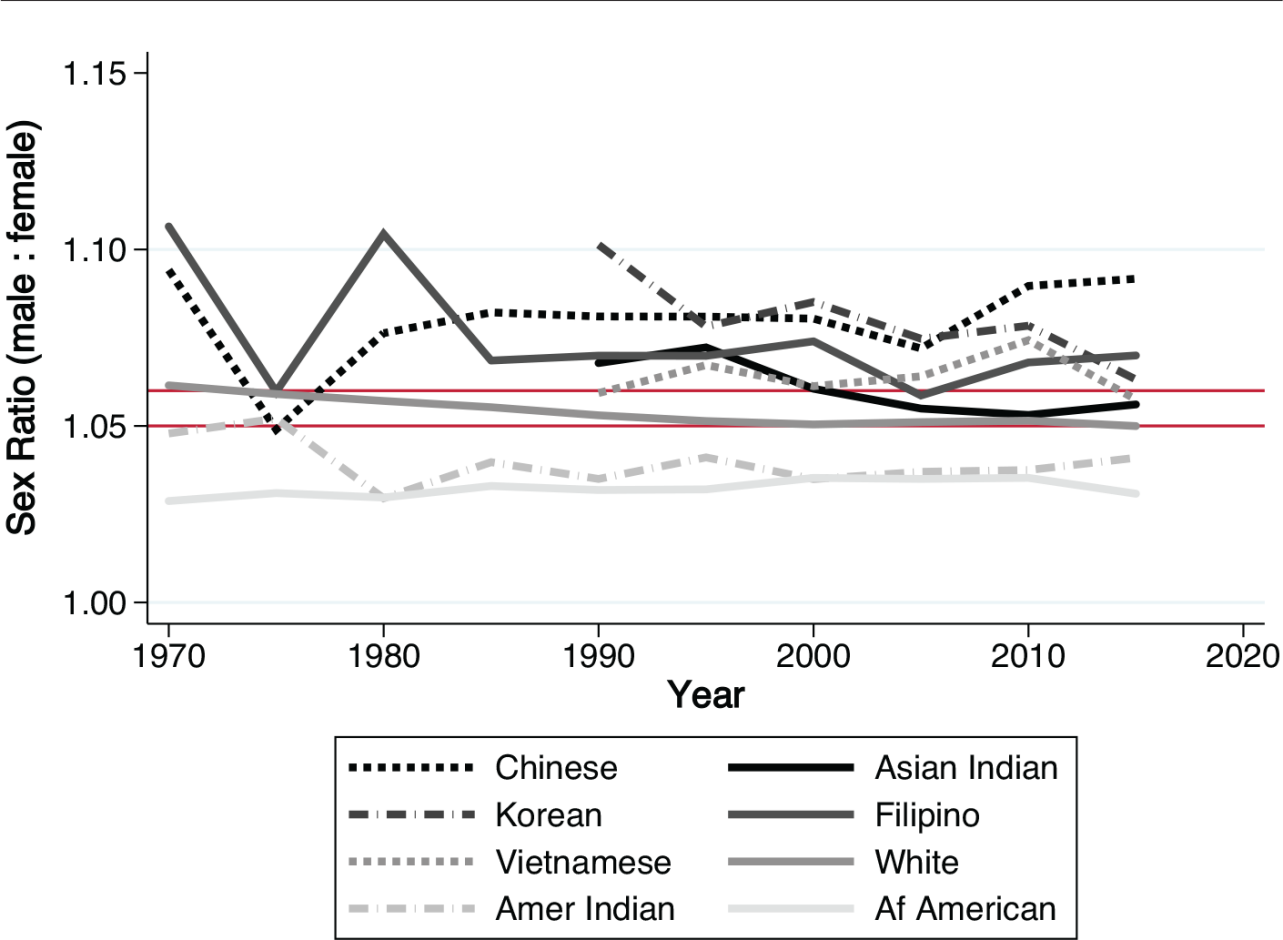
Trends in infant sex ratios by race/ethnicity in the United States for parities 1–3. Red horizontal rules indicate infant sex ratios within the biological norm. *Source:* NVSS 1969–2018, five-year means limited to births at parities 1–3 (live birth orders 1–3).

Figure 1 shows trends in infant sex ratios by maternal race/ethnicity from 1969 to 2018. The figure plots five-year mean values, weighted by the number of annual births in each race/ethnic category, because the small number of births for some categories can result in wide variation when using individual years. The infant sex ratios among parities 1–3 suggest son-biased ratios among Chinese, Asian Indian, Korean, Filipino, and Vietnamese births. However, ratios generally decline over time and, by the last period (2015–2018), Chinese and Filipino sex ratios remain elevated (at 1.09 and 1.07, respectively) but those for other groups decline to within or close to the biological norm. American Indian and African American ratios are consistently below the norm; White infant ratios are within the norm but decline over time.

**Fig. 2:**
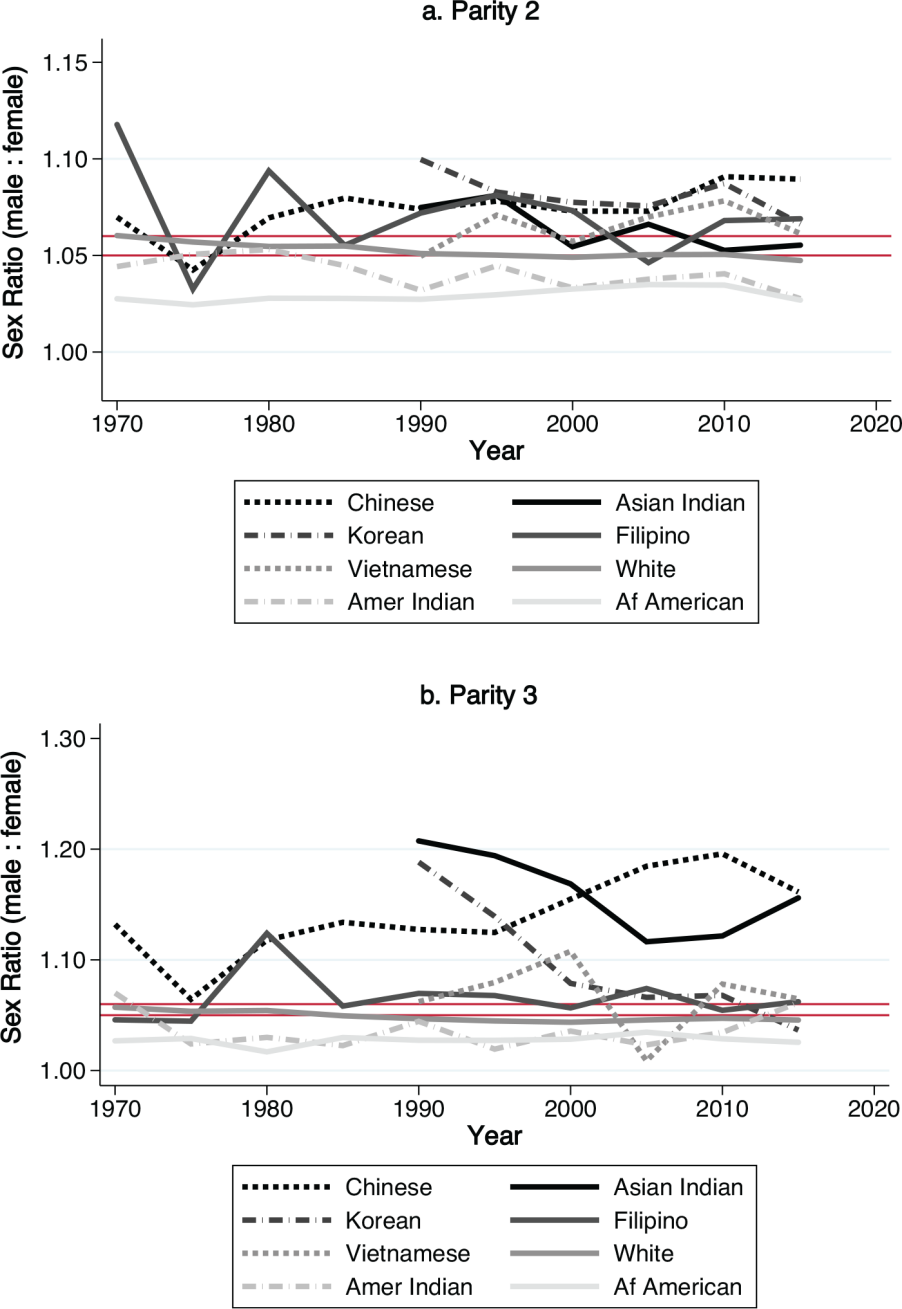
Trends in infant sex ratios by race/ethnicity in the United States for parity 2 (panel a) and parity 3 (panel b). Red horizontal rules indicate infant sex ratios within the biological norm. *Source:* NVSS 1969–2018, limited to births at parities 2 and 3.

Previous research has found that elevated sex ratios emerge at higher birth orders. Figure 2 shows infant sex ratios at second and third parity. Patterns among second births in panel a are similar to those when including births of parity 1–3. Male:female ratios are elevated in early years and decline over time, with the exception of Chinese ratios, which show a slight upward trend. Among third births (panel b), male-biased ratios of Chinese and Asian Indian births stand out from the other groups. Among Asian Indian births, the infant sex ratio declined from 1990 until about 2010, but increased after 2010. Ratios among Korean births also declined substantially after 1990 to the biological norm (or even slightly below) in the most recent period. This decline is consistent with a recent normalization trend found in South Korea (Chung and Das Gupta 2007). Ratios among Chinese births increased from 1975 to 2010 and declined (but remain elevated) in the most recent period.

Table A3 in the online appendix provides descriptive characteristics pooled across years by maternal education, race/ethnicity categories, and birth orders (1–3). Mean values by maternal education reveal slightly lower infant sex ratios among mothers with any college education. Regressions allow us to test whether this difference varies by race/ethnicity and whether it holds when controlling for stable differences between groups and time-varying economic and cultural characteristics.

**Fig. 3:**
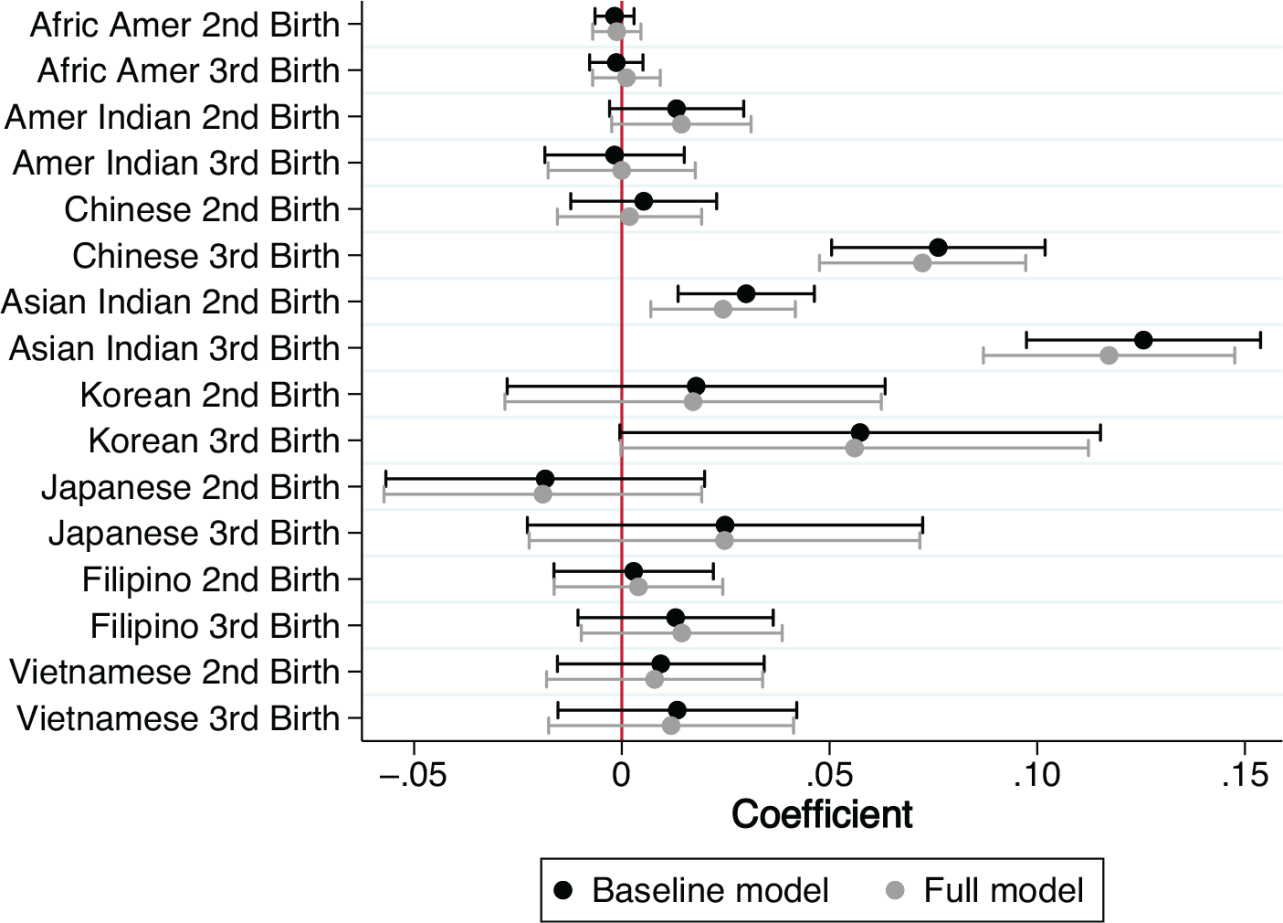
Coefficients predicting male:female infant sex ratios by parity and maternal race/ethnicity relative to White births. Estimates are from Models 1 and 5 in Table 1. The figure depicts coefficients for births at second and third parity (live birth orders 2 and 3) relative to first birth for each maternal race/ethnicity category (White is the omitted category). All models include indicators for each race/ethnicity category, birth order, and year. The full model includes controls for mean maternal and paternal age, marital status, proportion of mothers who live in the United States, proportions of births at first, second, and third parity, and aggregate group characteristics from the U.S. Census and ACS data measured separately by gender: proportions not in the labor force, foreign-born, living in a three-generation household, non-U.S. citizen, home ownership, Hispanic ethnicity, farm residence, and mean values of years living in the United States, family income, and home values. Whiskers represent 95% CIs. *Source:* NVSS 1969–2018.

Table 1 provides results from regression analyses predicting infant sex ratios at parities 1–3 with indicators for each maternal race/ethnicity category, birth order, and year. Figure 3 shows coefficients from these models predicting infant sex ratios at second and third parity by maternal race/ethnicity across all years. Estimates from the baseline model illustrate the average difference in infant sex ratios by parity for each group relative to the White ratio over all years. The full model includes controls for mean maternal and paternal age, marital status, proportion of mothers who live in the United States, proportions of births by parity in each race/ethnicity group (to adjust for declining fertility), and aggregate group characteristics from census and ACS data measured separately by gender.

Supporting Hypothesis 1a, results suggest that male:female ratios among births to Chinese and Asian Indian mothers are significantly higher (7–13%) than White ratios at third parity. Ratios are also slightly higher (1–3%) at second parity among these groups, but the difference is statistically significant only among Asian Indian births in the full models. The estimate is also high at third parity among births to Korean mothers, but is only significant at the 90% level. Contrary to Hypothesis 1b, the full model does not suggest significantly lower infant sex ratios among African American or American Indian births.

**Fig. 4:**
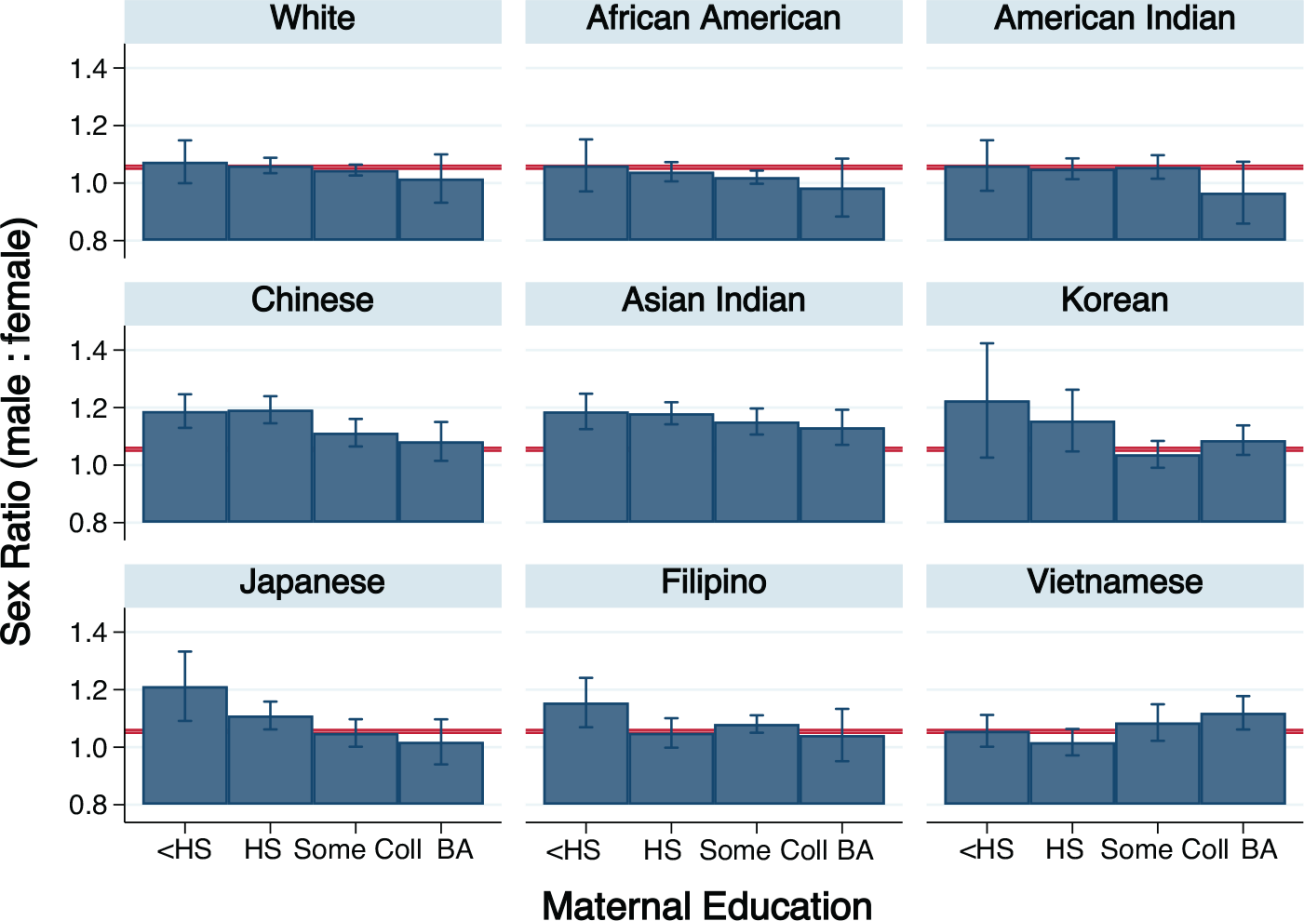
Predicted infant sex ratios by maternal education and race/ethnicity at parity 3. Red horizontal rules indicate infant sex ratios within the biological norm. The model includes indicators for each race/ethnicity category, maternal education category, and year and controls for mean maternal and paternal age, marital status, and proportion of mothers who live in the United States. Whiskers represent 95% CIs. HS = high school. BA = bachelor’s degree. *Source:* NVSS 1969–2018, limited to births at parity 3.

As found in previous work, results in Table 1 suggest that male-biased ratios are strongest at third parity. Table 2 provides predicted infant sex ratios at third parity by maternal race/ethnicity and education. For brevity, the table shows estimated differences for mothers with any college education compared to those with no college. Figure 4 illustrates average marginal predicted infant sex ratios by maternal education category (calculated using mean values of other variables) (Williams 2012). The main coefficient for college education is small and does not indicate a significant relationship to infant sex ratios among White births. In contrast, among Chinese and Japanese births, infant sex ratios are significantly lower at third parity when mothers have any college education. For example, infant sex ratios are about six percentage points lower among Chinese mothers with a college education (−0.07 + 0.01 = −0.06) compared to those with no college in the full model. With a total of about 112,000 third births to Chinese mothers and an average third-parity sex ratio of 1.15 during the time period examined, a six-percentage-point decrease in the sex ratio amounts to about 2,900 additional girls (60,000 boys/52,000 girls, increasing to 54,900 girls). This education difference is larger among Japanese mothers (nine percentage points). In the baseline model, the ratio is lower among Korean births when mothers have any college compared to those without, but these estimates are only marginally significant when including full controls.

Consistent with Hypothesis 2a, results suggest more balanced infant sex ratios when mothers have more education. The only exception is births to Vietnamese mothers, where predicted infant sex ratios are higher for births to mothers with college compared to those without. In comparing ratios among mothers with and without college (Table 2), evidence of greater equality at higher education is strongest among Chinese and Japanese births. When estimating variation by education level (Figure 4), male:female ratios are lower at higher education levels for nearly all groups in the baseline model. Particularly among mothers with a bachelor’s degree, predicted infant sex ratios are significantly lower in nearly all groups. Among mothers with a college degree, the 95% confidence interval for predicted ratios includes the normal range for all groups except in the case of Asian Indians.

While education is related to more balanced ratios for other groups, results in Table 2 suggest that sex ratios do not differ significantly by maternal education among African American and American Indian births. Thus, education may not sufficiently reduce exposure to environmental stressors to alter infant sex ratios (Feagin and Sikes 1995; Vines et al. 2006).

Table 3 shows estimated trends over time in sex ratios at third parity. Interaction terms between year and each race/ethnicity category in Model 1 suggest that male bias increased slightly over time among Chinese births and decreased among Korean births. However, these interaction terms become insignificant in the full model when controlling for economic and cultural measures. Thus, results are consistent with Hypothesis 3 without controls, but do not hold when including full control measures. This suggests that linear trends in infant sex ratios since 1969 are largely accounted for by time-varying changes, captured in our coarse measures of economic and cultural characteristics. Among Chinese births, however, the trend suggests a greater increase over time than among other groups, and this difference remains significant when including cultural or economic measures, but not when including both.

**Fig. 5:**
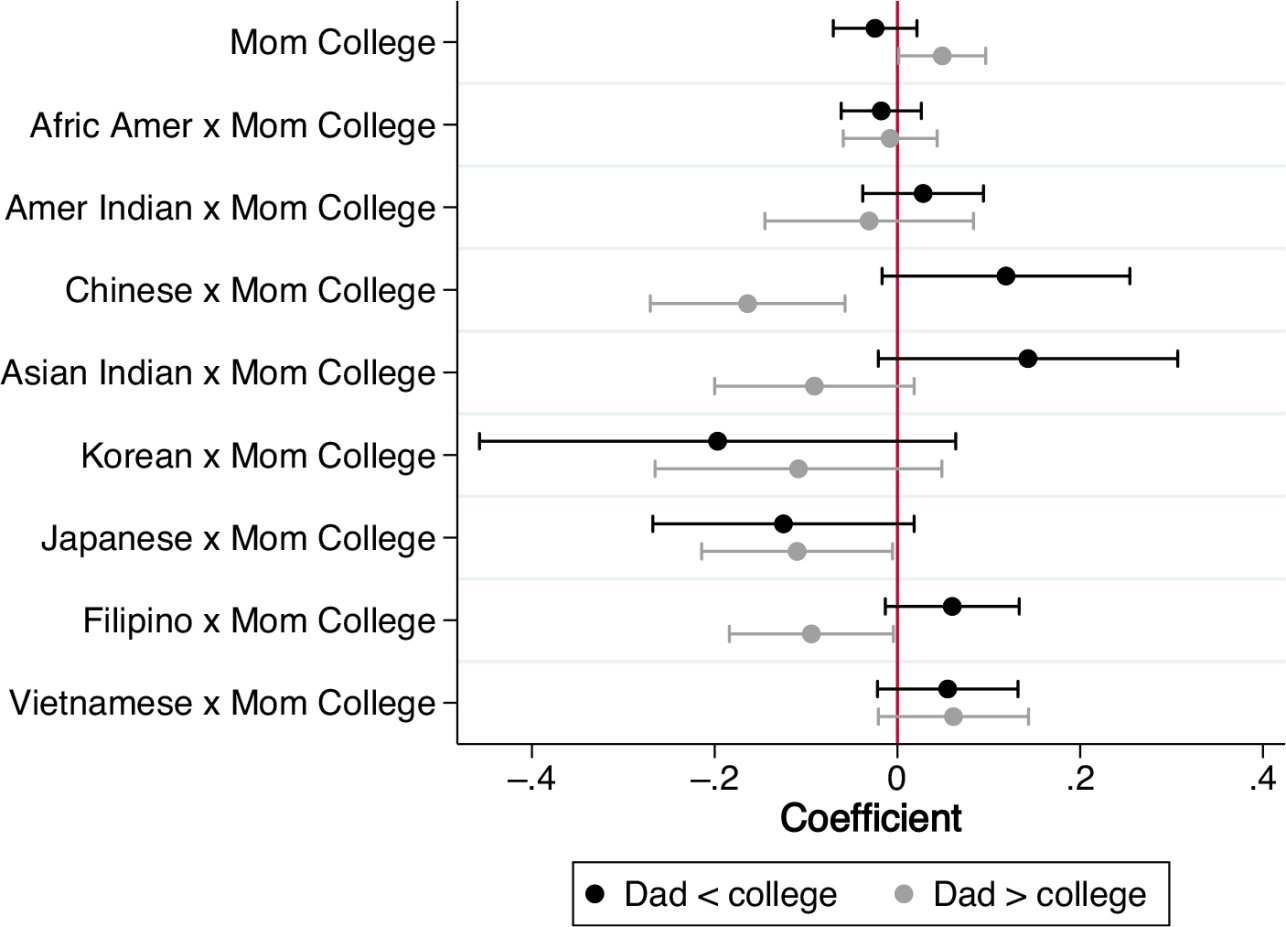
Coefficients for interaction between maternal race/ethnicity and indicator for maternal college education in separate models limited to births to fathers with or without college education. The models include indicators for each race/ethnicity category and year and controls for mean maternal and paternal age, marital status, proportion of mothers who live in the United States, proportions of births at first, second, and third parity, and aggregate group characteristics from the U.S. Census and ACS data measured separately by gender: proportions not in the labor force, foreign-born, living in a three-generation household, non-U.S. citizen, home ownership, Hispanic ethnicity, farm residence, and mean values of years living in the United States, family income, and home values. Whiskers represent 95% CIs. *Source:* NVSS 1969–2018, limited to births at parities 2–3.

Table 4 provides estimates of variation in the relationship between maternal education and infant sex ratios at second and third parity by paternal education. Estimates use Eq. (1) and interact maternal race/ethnicity categories with education categories. For Chinese, Asian Indian, and Filipino births, estimates suggest a more equalizing role of maternal college education when the father has also attended college. Shaded cells indicate that coefficients for maternal college education differ significantly between models by paternal education (Paternoster et al. 1998). For example, compared to Chinese mothers with no college education, college-educated Chinese mothers have marginally more male-biased ratios when the father has not attended college and significantly less biased ratios when the father has attended college. Figure 5 illustrates the estimated relationship between maternal college education and infant sex ratios by race/ethnicity when the father has or has not attended college. Consistent with Hypothesis 4, maternal college education is related to more equal infant sex ratios when both parents have attended college. However, these estimates are only significant among Chinese, Japanese, and Filipino births, and the coefficients rarely differ significantly by paternal education. Shaded cells in Table 4 indicate that the equality benefits of maternal education among Chinese and Filipino births are significantly larger when the father also attended college.

### Sensitivity Analyses

Tables A4–A6 in the online appendix show results predicting mean live birth order as a rough measure of fertility. Table A4 indicates variation by maternal race/ethnicity, with significantly higher mean birth order among births to American Indians and significantly lower mean birth orders among births to Chinese, Asian Indian, Korean, Japanese, Filipino, and Vietnamese, compared with Whites. These differences remain significant across all models. Table A5 suggests variation in fertility by education within maternal race/ethnicity category. Mean birth order is significantly lower among college-educated White mothers. Birth orders are even lower, however, among college-educated African American, American Indian, Chinese, and Asian Indian mothers (and marginally lower among college-educated Vietnamese mothers), compared to their counterparts who have not attended college. The only positive coefficient for college education—for Filipino births—is similar in size to the main coefficient for college education and suggests no difference in fertility by maternal college education among Filipino births. Examining variation over time, Table A6 suggests significant declines in mean birth order since 1970 among Chinese, Filipino, and Vietnamese mothers. Thus, results are somewhat consistent with declining fertility (Hypothesis 3). This pattern is consistent with previous evidence of lower fertility at higher education (Almond et al. 2013; Lesthaeghe 2010). However, more balanced infant sex ratios at higher education do not support the argument that education increases sex selection by reducing fertility.

Tables A7–A9 in the online appendix show estimates predicting inequality of prenatal care. Estimates in the full model in Table A7 suggest more prenatal visits during pregnancies with male compared to female infants at third parity among Asian Indian and Japanese births. Estimates are also elevated, but only marginally significant, among third Filipino and Vietnamese births. Estimates in Table A8 suggest more prenatal visits when pregnant with sons among African American, Asian Indian, Filipino, and Vietnamese mothers with less than college education. However, prenatal visits are significantly more balanced by infant sex among college-educated mothers in each of these groups. Estimates in Table A9 suggest an overall trend toward increasing equality of prenatal visits by infant sex across all groups, but little evidence of variation by maternal race/ethnicity. Overall, results for prenatal care are consistent with the main analyses: more balanced prenatal care over time and with higher maternal education. However, results using this less extreme measure of gender inequality are not as strong as results for infant sex ratios.

Estimates may vary by racial/ethnic identification of the father. We repeat analyses examining variation by paternal race/ethnicity and when limited to parents of the same race/ethnic identity. Results are consistent with the main analyses.

The most elevated child sex ratios emerge when examining variation at high parities by the sex of previous children. NVSS data do not allow analyses conditional on the sex of previous births because the data do not include that information. Sensitivity analyses (discussed in the Supplementary Online Appendix) use American Community Survey data for 2000–2018 to examine child sex ratios by parental race/ethnicity and education, conditional on the sex of previous children. Results are consistent with those using NVSS data and suggest greater equality at higher levels of parental education.

## Discussion

We examine gender bias in the ratio of live births using administrative birth certificate data from NVSS from 1969 to 2018. Building on existing work, we assess trends over five decades in infant sex ratios by birth parity and across multiple racial/ethnic categories. Examining infant sex ratios, a measure that is not susceptible to social desirability bias, we find that gender status equality has improved for many groups, but progress varies by racial/ethnic background and by education. This suggests that the “stalled gender revolution” (England 2010; England et al. 2020) holds for status among Chinese and Asian Indian parents, with less progress toward gender equality in status in recent decades. Results provide further evidence of son-biased infant sex ratios among third births to Chinese and Asian Indian mothers in the United States. Even unconditional on sex of the previous child, male bias is also evident among second births to Asian Indian mothers.

Contrary to evidence from China (Almond et al. 2019), regression analyses indicate greater equality of infant sex ratios when mothers have more education. Ratios are particularly more equal among mothers with a bachelor’s degree. Predicted infant sex ratios do not differ from the biological norm among third births to Chinese or Korean mothers when the mother has completed college. In fact, only among Asian Indian mothers do predicted infant sex ratios differ from the biological norm among mothers with a college degree.

By examining heterogeneity by education among a more diverse set of racial/ethnic groups in more recent years than in previous research, our results identify additional ethnic and racial groups with sex ratios that differ from the biological norm. For example, we find that infant sex ratios are lower among African American and American Indian births, as predicted based on greater exposure to pollution and chronic socioeconomic stress (Figá-Talamanca and Petrelli 2000; Goldsmith et al. 1984; Sakamoto et al. 2001; Whorton et al. 1994). However, our finding that sex ratios do not differ significantly by maternal education among African American and American Indian births suggests that education may not sufficiently reduce exposure to environmental stressors to alter infant sex ratios (Feagin and Sikes 1995; Vines et al. 2006). Consistent with Abrevaya (2009), we find that infant sex ratios are more strongly related to education among Chinese than among Asian Indian Americans. A novel finding is male-biased sex ratios among Japanese and Filipino mothers with less than a high school education. The educational gradient of sex ratios at third parity among Japanese and Filipinos is similar to that among Chinese births. Births to Vietnamese mothers also warrant additional attention, given evidence of heightened sex ratios at higher levels of education (Guilmoto 2009).

Education is more strongly related to balanced child sex ratios when both parents have attended college, providing further evidence that parental education is related to more balanced infant sex ratios. Estimates hold when controlling for aggregate economic measures, which is not consistent with the theory that education increases male-biased ratios by increasing access to sex-selective technologies. Rather, results are more consistent with the possibility that education alters preferences for male children by shaping cultural preferences (Baker 2014).

Our analyses include controls for aggregate cultural and economic measures, however, our reliance on aggregate rather than individual economic and cultural measures is a key limitation. Future research with parental cultural and economic information could provide a stronger test of when parental preferences change and how that relates to infant sex ratios. Descriptive trends document greater equality of infant sex ratios over time for most groups, but trend estimates are not significant when controlling for aggregate measures of both economic and cultural characteristics. Future studies using finer measures are warranted.

A second limitation of our study is that NVSS data do not allow examination of variation by the sex of previous children. We use an alternative data source (pooled ACS data for 2000–2018) to address this and to examine variation in child sex ratios by parental education and race/ethnicity conditional on the sex of previous children. These analyses are discussed in the Supplementary Online Appendix, and those results are consistent with our main analyses.

A third limitation is that our focus on the sex ratio of live births could miss less extreme bias, including inequality of prenatal health among live births. Gender bias could influence infant health if mothers adjust prenatal behaviors depending on the sex of the fetus. We examine variation in prenatal care by infant sex and find results to be broadly consistent with the main analyses: more balanced prenatal care over time and with higher maternal education. However, results using this less extreme measure of gender inequality are not as strong as results for infant sex ratios. Consistent with recent evidence from the United States (Almond and Cheng 2021; Almond and Sun 2017; Muchomba and Chatterji 2020), we find higher prenatal care for boys among Asian Indian births, with more equal prenatal care at higher levels of maternal education. Building on this work, we also find some evidence of this relationship among additional groups: African American, Japanese, Filipino, and Vietnamese mothers. However, these differences do not always hold in models with full controls.

Developing countries have sought to increase equality in child sex ratios through economic and cultural interventions (e.g., media campaigns) or through abortion restrictions (Chung and Das Gupta 2007; Jimmerson 1990; Rebouche 2015; Sadh and Kapoor 2012). Evidence suggests that efforts to limit reproductive rights in the United States could exacerbate gender inequality, particularly among immigrant and nonmajority ethnic groups, partially owing to heightened discrimination and social sanctions (Barot 2012; Mussino et al. 2018). We find that infant sex ratios are more equal when both parents have a college education. Our results are associational and cannot address potentially unequal selection into higher education by child sex preferences. However, we examine the relationship within racial/ethnic groups, which addresses stable differences between groups. In contrast to evidence from China (Almond et al. 2019), our findings are not consistent with increased sex-selective practices at higher levels of education. If our results are not fully explained by selection into education, then higher education expansion in recent cohorts (Averett and Burton 1996) could yield more balanced sex ratios and greater equality in sex preferences in younger families. Overall, our findings suggest that higher education is related to more equal gender status in the United States.

## Supplementary Material

Supplement

## Figures and Tables

**Table 1:** Predicted infant sex ratios by birth order and maternal race/ethnicity

	Infant Male:Female Ratio				
Variable	(1)	(2)	(3)	(4)	(5)

African American	−0.021**	−0.038**	−0.073^†^	−0.050^†^	−0.049
	(0.002)	(0.013)	(0.041)	(0.030)	(0.075)
American Indian	−0.014**	−0.023*	−0.017	−0.040*	−0.018
	(0.005)	(0.010)	(0.014)	(0.017)	(0.033)
Chinese	0.019**	0.015	−0.083	−0.014	−0.008
	(0.007)	(0.009)	(0.065)	(0.025)	(0.117)
Asian Indian	−0.012^†^	−0.011	−0.129^†^	−0.047	−0.078
	(0.007)	(0.013)	(0.067)	(0.031)	(0.122)
Korean	0.019	0.017	−0.093	−0.037	−0.038
	(0.014)	(0.016)	(0.062)	(0.033)	(0.100)
Japanese	0.024*	0.021	−0.040	−0.014	−0.014
	(0.012)	(0.014)	(0.045)	(0.026)	(0.062)
Filipino	0.020**	0.013	−0.059	0.005	0.010
	(0.007)	(0.009)	(0.072)	(0.027)	(0.122)
Vietnamese	0.007	−0.001	−0.096	−0.012	−0.015
	(0.009)	(0.015)	(0.076)	(0.021)	(0.130)
Birth Order 2	−0.005**	−0.005	−0.006	−0.005	−0.005
	(0.002)	(0.003)	(0.004)	(0.004)	(0.005)
Birth Order 3	−0.008**	−0.012	−0.014^†^	−0.014^†^	−0.014
	(0.002)	(0.008)	(0.008)	(0.008)	(0.010)
African American, Birth Order 2	−0.002	−0.002	−0.001	−0.001	−0.001
	(0.003)	(0.003)	(0.004)	(0.004)	(0.004)
African American, Birth Order 3	−0.001	−0.000	0.001	0.001	0.001
	(0.003)	(0.004)	(0.004)	(0.004)	(0.004)
American Indian, Birth Order 2	0.013	0.014	0.014^†^	0.014^†^	0.014
	(0.008)	(0.009)	(0.009)	(0.008)	(0.009)
American Indian, Birth Order 3	−0.002	−0.000	−0.000	0.000	−0.000
	(0.011)	(0.011)	(0.011)	(0.011)	(0.011)
Chinese, Birth Order 2	0.005	0.003	0.002	0.002	0.002
	(0.010)	(0.010)	(0.010)	(0.010)	(0.010)
Chinese, Birth Order 3	0.076**	0.073**	0.072**	0.072**	0.072**
	(0.012)	(0.012)	(0.012)	(0.012)	(0.012)
Asian Indian, Birth Order 2	0.030**	0.025**	0.024*	0.024*	0.024*
	(0.009)	(0.010)	(0.010)	(0.010)	(0.010)
Asian Indian, Birth Order 3	0.126**	0.118**	0.117**	0.117**	0.117**
	(0.014)	(0.015)	(0.016)	(0.015)	(0.016)
Korean, Birth Order 2	0.018	0.018	0.017	0.017	0.017
	(0.021)	(0.021)	(0.021)	(0.021)	(0.021)
Korean, Birth Order 3	0.057^†^	0.057^†^	0.057^†^	0.056^†^	0.056^†^
	(0.030)	(0.030)	(0.030)	(0.030)	(0.030)
Japanese, Birth Order 2	−0.018	−0.019	−0.019	−0.019	−0.019
	(0.017)	(0.018)	(0.018)	(0.017)	(0.017)
Japanese, Birth Order 3	0.025	0.025	0.025	0.025	0.025
	(0.022)	(0.022)	(0.022)	(0.022)	(0.022)
Filipino, Birth Order 2	0.003	0.004	0.004	0.004	0.004
	(0.010)	(0.010)	(0.010)	(0.010)	(0.010)
Filipino, Birth Order 3	0.013	0.015	0.015	0.015	0.014
	(0.013)	(0.013)	(0.013)	(0.013)	(0.013)
Vietnamese, Birth Order 2	0.009	0.009	0.008	0.008	0.008
	(0.012)	(0.013)	(0.013)	(0.013)	(0.013)
Vietnamese, Birth Order 3	0.013	0.013	0.012	0.012	0.012
	(0.016)	(0.016)	(0.016)	(0.016)	(0.016)
Constant	1.063**	0.616	0.493	0.459	0.406
	(0.024)	(1.475)	(1.542)	(1.465)	(1.528)
Observations	4,571	4,571	4,571	4,571	4,571
*R* ^2^	.063	.065	.068	.068	.071
Race/Ethnicity, Birth Order, and Year Indicators	Y	Y	Y	Y	Y
Controls for Parental Characteristics		Y	Y	Y	Y
Controls for Cultural Measures			Y		Y
Controls for Economic Measures				Y	Y

*Notes:* All models include indicators for each race/ethnicity category, birth order, and year. The full model includes controls for mean maternal and paternal age, marital status, proportion of mothers who live in the United States, proportions of births at first, second, and third parity, and aggregate group characteristics from the U.S. Census and ACS data measured separately by gender: proportions not in the labor force, foreign-born, living in a three-generation household, non-U.S. citizen, home ownership, Hispanic ethnicity, farm residence, and mean values of years living in the United States, family income, and home values. Bootstrapped standard errors stratified by race/ethnicity are shown in parentheses.

*Source:* NVSS 1969–2018, limited to births at parities 1–3 (live birth orders 1–3).

† *p* < .10

* *p* < .05

** *p* < .01

**Table 2:** Predicted infant sex ratios at third parity by maternal race/ethnicity and education

	Infant Male:Female Ratio				
Variable	(1)	(2)	(3)	(4)	(5)

African American	−0.019**	−0.052	0.008	−0.114	0.091
	(0.006)	(0.033)	(0.090)	(0.072)	(0.159)
American Indian	−0.015^†^	−0.030	−0.015	−0.075^†^	−0.021
	(0.008)	(0.019)	(0.032)	(0.044)	(0.075)
Chinese	0.132**	0.122**	−0.026	0.059	0.348
	(0.019)	(0.036)	(0.154)	(0.067)	(0.303)
Asian Indian	0.123**	0.114**	−0.054	0.111	0.343
	(0.019)	(0.044)	(0.156)	(0.074)	(0.316)
Korean	0.128*	0.118*	−0.112	0.019	0.198
	(0.051)	(0.059)	(0.158)	(0.084)	(0.285)
Japanese	0.096**	0.092*	−0.033	0.035	0.111
	(0.031)	(0.038)	(0.118)	(0.070)	(0.181)
Filipino	0.035*	0.028	−0.063	−0.030	0.298
	(0.015)	(0.021)	(0.168)	(0.074)	(0.316)
Vietnamese	−0.018	−0.037	−0.169	−0.076	0.252
	(0.016)	(0.035)	(0.177)	(0.052)	(0.324)
Mother Has Any College Education	0.007	0.010	0.010	0.012	0.014
	(0.006)	(0.017)	(0.016)	(0.016)	(0.016)
African American × Mom Any College	−0.007	0.006	0.009	0.008	0.017
	(0.010)	(0.016)	(0.018)	(0.017)	(0.023)
American Indian × Mom Any College	−0.002	0.000	0.002	0.002	0.007
	(0.019)	(0.019)	(0.020)	(0.020)	(0.024)
Chinese × Mom Any College	−0.074**	−0.078**	−0.077**	−0.079**	−0.072*
	(0.026)	(0.029)	(0.029)	(0.030)	(0.033)
Asian Indian × Mom Any College	−0.031	−0.027	−0.024	−0.027	−0.022
	(0.026)	(0.027)	(0.027)	(0.027)	(0.038)
Korean × Mom Any College	−0.114*	−0.107^†^	−0.103^†^	−0.105*	−0.103^†^
	(0.053)	(0.055)	(0.054)	(0.054)	(0.057)
Japanese × Mom Any College	−0.096**	−0.099**	−0.101**	−0.099**	−0.105**
	(0.035)	(0.038)	(0.039)	(0.039)	(0.041)
Filipino × Mom Any College	−0.005	−0.007	−0.006	−0.007	−0.004
	(0.019)	(0.020)	(0.021)	(0.020)	(0.021)
Vietnamese × Mom Any College	0.066**	0.075*	0.080**	0.076*	0.091**
	(0.025)	(0.029)	(0.030)	(0.030)	(0.032)
Constant	1.046**	0.721	0.053	0.039	−0.232
	(0.044)	(2.799)	(2.587)	(2.803)	(2.707)
Observations	1,523	1,523	1,523	1,523	1,523
*R* ^2^	.120	.122	.130	.128	.137
Race/Ethnicity, Education, and Year Indicators	Y	Y	Y	Y	Y
Controls for Parental Characteristics		Y	Y	Y	Y
Controls for Cultural Measures			Y		Y
Controls for Economic Measures				Y	Y

*Notes:* All models include indicators for each race/ethnicity category, maternal college education, and year. The full model includes controls for mean maternal and paternal age, marital status, proportion of mothers who live in the United States, proportions of births at first, second, and third parity, and aggregate group characteristics from the U.S. Census and ACS data measured separately by gender: proportions not in the labor force, foreign-born, living in a three-generation household, non-U.S. citizen, home ownership, Hispanic ethnicity, farm residence, and mean values of years living in the United States, family income, and home values. Bootstrapped standard errors stratified by race/ethnicity are shown in parentheses.

*Source:* NVSS 1969–2018, limited to births at parity 3 (live birth order 3).

† *p* < .10

* *p* < .05

** *p* < .01

**Table 3:** Predicted infant sex ratios at third parity by maternal race/ethnicity and year

	Infant Male:Female Ratio				
Variable	(1)	(2)	(3)	(4)	(5)

African American	−0.933	−0.581	−2.465	−1.644	7.184
	(0.644)	(0.865)	(4.602)	(2.230)	(7.915)
American Indian	−2.075	−1.604	−1.087	−2.086	1.732
	(1.549)	(1.715)	(2.785)	(1.904)	(5.499)
Chinese	−5.427**	−6.145**	−7.141*	−11.293**	−7.517
	(1.873)	(1.982)	(3.361)	(3.629)	(6.804)
Asian Indian	6.326*	5.358	4.606	1.551	6.117
	(3.196)	(3.499)	(5.086)	(5.359)	(9.054)
Korean	12.656**	12.724**	12.575^†^	1.784	15.013
	(4.740)	(4.704)	(6.838)	(7.311)	(12.752)
Japanese	1.000	0.601	−3.361	−2.064	7.044
	(2.488)	(2.554)	(9.765)	(4.757)	(13.056)
Filipino	0.267	0.171	−0.501	−2.682	1.823
	(1.662)	(1.686)	(4.315)	(3.684)	(7.160)
Vietnamese	4.879	5.338	6.382	0.060	4.038
	(3.415)	(3.772)	(8.515)	(7.094)	(13.474)
Year	−0.001	−0.001	−0.002	−0.004	0.003
	(0.001)	(0.002)	(0.003)	(0.003)	(0.004)
African American × Year	0.000	0.000	0.001	0.001	−0.004
	(0.000)	(0.000)	(0.002)	(0.001)	(0.004)
American Indian × Year	0.001	0.001	0.001	0.001	−0.001
	(0.001)	(0.001)	(0.001)	(0.001)	(0.003)
Chinese × Year	0.003**	0.003**	0.004*	0.006**	0.004
	(0.001)	(0.001)	(0.002)	(0.002)	(0.003)
Asian Indian × Year	−0.003^†^	−0.003	−0.002	−0.001	−0.003
	(0.002)	(0.002)	(0.002)	(0.003)	(0.004)
Korean × Year	−0.006**	−0.006**	−0.006^†^	−0.001	−0.007
	(0.002)	(0.002)	(0.003)	(0.004)	(0.006)
Japanese × Year	−0.000	−0.000	0.002	0.001	−0.003
	(0.001)	(0.001)	(0.005)	(0.002)	(0.007)
Filipino × Year	−0.000	−0.000	0.000	0.001	−0.001
	(0.001)	(0.001)	(0.002)	(0.002)	(0.004)
Vietnamese × Year	−0.002	−0.003	−0.003	−0.000	−0.002
	(0.002)	(0.002)	(0.004)	(0.004)	(0.007)
Constant	2.307	2.532	4.831	8.471	−5.090
	(1.959)	(4.770)	(6.525)	(7.006)	(8.949)
Observations	1,523	1,523	1,523	1,523	1,523
*R* ^2^	.109	.113	.114	.116	.127
Race/Ethnicity and Year Indicators	Y	Y	Y	Y	Y
Controls for Parental Characteristics		Y	Y	Y	Y
Controls for Cultural Measures			Y		Y
Controls for Economic Measures				Y	Y

*Notes:* All models include indicators for each race/ethnicity category and year. The full model includes controls for mean maternal and paternal age, marital status, proportion of mothers who live in the United States, proportions of births at first, second, and third parity, and aggregate group characteristics from the U.S. Census and ACS data measured separately by gender: proportions not in the labor force, foreign-born, living in a three-generation household, non-U.S. citizen, home ownership, Hispanic ethnicity, farm residence, and mean values of years living in the United States, family income, and home values. Bootstrapped standard errors stratified by race/ethnicity are shown in parentheses.

*Source:* NVSS 1969–2018, limited to births at parity 3 (live birth order 3).

† *p* < .10

* *p* < .05

** *p* < .01

**Table 4:** Predicted infant sex ratios by race/ethnicity and parental education

	Infant Male:Female Ratio			
	Dad < College		Dad ≥ College	
Variable	(1)	(2)	(3)	(4)

African American	−0.016**	−0.183	0.005	−0.943
	(0.005)	(0.776)	(0.010)	(0.730)
American Indian	−0.008	−0.106	0.147**	−0.380
	(0.009)	(0.472)	(0.040)	(0.435)
Chinese	0.110**	0.206	0.213**	−0.727
	(0.028)	(0.936)	(0.043)	(0.752)
Asian Indian	0.109**	0.590	0.160**	−0.476
	(0.030)	(0.719)	(0.051)	(0.633)
Korean	0.339**	0.740	0.158*	−0.697
	(0.117)	(0.919)	(0.077)	(0.708)
Japanese	0.218**	0.264	0.109**	−0.280
	(0.047)	(0.364)	(0.037)	(0.286)
Filipino	0.070**	0.558	0.144**	−0.959
	(0.019)	(0.968)	(0.043)	(0.793)
Vietnamese	0.037*	0.541	−0.004	−1.194
	(0.022)	(1.149)	(0.028)	(0.911)
Mother Has Any College Education	0.005	−0.024	0.007	0.049*
	(0.005)	(0.023)	(0.007)	(0.024)
African American × Mom Any College	−0.011	−0.018	−0.031**	−0.008
	(0.010)	(0.022)	(0.012)	(0.026)
American Indian × Mom Any College	0.030	0.028	−0.063	−0.031
	(0.028)	(0.034)	(0.070)	(0.058)
Chinese × Mom Any College	0.079	0.119^†^	−0.129**	−0.164**
	(0.053)	(0.069)	(0.050)	(0.054)
Asian Indian × Mom Any College	0.118	0.143^†^	−0.059	−0.091
	(0.082)	(0.084)	(0.054)	(0.056)
Korean × Mom Any College	−0.233^†^	−0.197	−0.088	−0.108
	(0.132)	(0.133)	(0.082)	(0.080)
Japanese × Mom Any College	−0.133^†^	−0.125^†^	−0.079^†^	−0.110*
	(0.069)	(0.073)	(0.042)	(0.053)
Filipino × Mom Any College	0.058	0.060	−0.094^†^	−0.094*
	(0.036)	(0.037)	(0.052)	(0.046)
Vietnamese × Mom Any College	0.018	0.055	0.078^†^	0.061
	(0.041)	(0.039)	(0.040)	(0.042)
Constant	1.127**	−0.880	1.153**	4.030
	(0.052)	(1.856)	(0.063)	(2.515)
Observations	3,634	3,632	3,733	3,732
*R* ^2^	.044	.063	.036	.045
Race/Ethnicity, Education, and Year Indicators	Y	Y	Y	Y
Parental, Cultural, and Economic Controls		Y		Y

*Notes:* Models 1 and 2 are limited to fathers with no college education. Models 3 and 4 are limited to fathers with at least some college education. Shaded cells indicate significant difference (*p* < .05) between maternal education coefficients by paternal education (Models 1 vs. 3 and 2 vs. 4). All models include indicators for each race/ethnicity category, maternal college education, and year. The full model includes controls for mean maternal and paternal age, marital status, proportion of mothers who live in the United States, proportions of births at first, second, and third parity, and aggregate group characteristics from the U.S. Census and ACS data measured separately by gender: proportions not in the labor force, foreign-born, living in a three-generation household, non-U.S. citizen, home ownership, Hispanic ethnicity, farm residence, and mean values of years living in the United States, family income, and home values. Bootstrapped standard errors stratified by race/ethnicity are shown in parentheses.

*Source:* NVSS 1969–2018, limited to births at parities 2–3.

† *p* < .10

* *p* < .05

** *p* < .01
