# Supplementary material for: Learning to Value Girls: Balanced Infant Sex Ratios at Higher Parental Education in the United States, 1969–2018
Source: Demography. Author manuscript; Available in PMC 2023 Jun 1. (PMC9791645; doi:10.1215/00703370-9968420)
Supplement: Supplement [file NIHMS1856062-supplement-Supplement.pdf]

Learning to Value Girls: Balanced Infant Sex Ratios at Higher Parental Education in the U.S. 1969-2018

Emily Rauscher and Haoming Song

Appendix A

Table A1: Proportion of Birth Records Missing Information

Variable	Proportion Missing			
	1969-2018	Excluding 1995-2010 without Paternal Education		
	All Records	All	Records with Paternal Education	Records Missing Paternal Education
Maternal Education	0.107	0.141	0.002	0.528
Maternal Race/Ethnicity	0.006	0.003	0.001	0.008
Maternal Age	0.000	0.001	0.000	0.002
Paternal Education	0.533	0.263	0.000	1.000
Paternal Race/Ethnicity	0.153	0.142	0.022	0.477
Paternal Age	0.140	0.140	0.008	0.509
Married	0.051	0.080	0.062	0.131
Mom US Resident	0.000	0.001	0.000	0.003
Birth Order	0.007	0.008	0.005	0.015
Infant Sex	0.000	0.001	0.000	0.002
Prenatal Visits	0.080	0.107	0.032	0.032
Birth Weight	0.001	0.002	0.001	0.003
Gestational Length	0.063	0.096	0.063	0.187
N Birth Records (1,000s)	177109.30	112299.33	82784.07	29515.26

NVSS 1969-2018. Values indicate the proportion of birth records missing information for each variable. The first column includes all years (1969-2018). The other columns include 1969-1994 and 2011-2018, years with paternal education information. The total number of birth records in all years is higher than the number used in this paper because they include birth orders higher than 3 and racial/ethnic groups not examined here (e.g., Guamanian, Samoan). Records with paternal education have non-missing paternal education.

Table A2: Data Source for Aggregate Values Linked to Each Year of Infant Sex Ratios

Birth Years	Aggregate Data Source
1969-1974	1970 Census
1975-1984	1980 Census
1985-1994	1990 Census
1995-2000	2000 Census
2001-2005	2001-2005 ACS Annual Samples
2006-2010	2006-2010 ACS 5-year Sample
2011-2015	2011-2015 ACS 5-year Sample
2016-2018	2014-2018 ACS 5-year Sample

Table A3: Descriptive Statistics

Variable	Mean	Std Dev	No College	Any College
Infant Male:Female Ratio	1.07	0.13	1.07	1.06
White	0.13	0.34	0.13	0.13
African American	0.13	0.34	0.13	0.13
American Indian	0.13	0.34	0.13	0.13
Chinese	0.13	0.34	0.13	0.13
Asian Indian	0.07	0.26	0.07	0.07
Korean	0.07	0.26	0.07	0.07
Japanese	0.13	0.34	0.13	0.13
Filipino	0.13	0.34	0.13	0.13
Vietnamese	0.07	0.26	0.07	0.07
<HS	0.25	0.43	0.50	-
HS	0.25	0.43	0.50	-
Some Coll	0.25	0.43	-	0.50
BA	0.25	0.43	-	0.50
Year	1995.95	14.11	1995.95	1995.95
Maternal Age	28.18	3.93	26.24	30.11
Paternal Age	31.44	3.73	30.11	32.78
Married	0.75	0.24	0.64	0.86
Mom US Resident	1.00	0.00	1.00	1.00
Proportion Birth Order 1	0.42	0.06	0.39	0.45
Proportion Birth Order 2	0.33	0.05	0.31	0.35
Proportion Birth Order 3	0.15	0.03	0.16	0.13
Mean Birth Order	2.00	0.30	2.16	1.85
Number of Births	29979.42	72553.20	32371.64	27587.21
Aggregate Census & ACS Measures				
% Not in Labor Force - Women	0.35	0.08	0.35	0.35
% Not in Labor Force - Women	0.22	0.06	0.22	0.22
% Foreign Born - Women	0.47	0.34	0.00	0.00
% Foreign Born - Men	0.44	0.33	0.47	0.47
% 3 Generation Hh - Women	0.10	0.04	0.44	0.44
% 3 Generation Hh - Men	0.09	0.04	0.09	0.09
Mean Years in the US - Women	16.98	16.50	16.98	16.98
Mean Years in the US - Men	19.18	24.85	19.18	19.18
% Non-Citizen - Women	0.25	0.19	0.25	0.25
% Non-Citizen - Men	0.24	0.18	0.24	0.24
% Home Ownership - Women	0.57	0.08	0.57	0.57
% Home Ownership - Men	0.57	0.08	0.57	0.57
Mean Home Value - Women	401944.60	142271.90	401944.60	401944.60

Mean Home Value - Men	405425.30	146591.00	405425.30	405425.30
% Hispanic - Women	0.04	0.05	0.04	0.04
% Hispanic - Men	0.05	0.06	0.05	0.05
Mean Family Income - Women	97180.73	24804.11	97180.73	97180.73
Mean Family Income - Men	97188.08	21696.10	97188.08	97188.08
% Farm Residence - Women	0.01	0.01	0.01	0.01
% Farm Residence - Men	0.01	0.01	0.01	0.01
N Race/Ethnic-Birth Order-Education Category-Year	4,572		2,286	2,286

NVSS 1969-2018, limited to births at parities 1-3 (live birth orders 1-3). Linked by year and race/ethnic category to aggregate Census and ACS data 1970-2018.

Table A4: Predicted Mean Live Birth Order by Maternal Race/Ethnicity

	(1)	(2)	(3)	(4)	(5)
VARIABLES			Mean Live Birth Order		
African American	0.133**	-0.101**	0.110*	-0.309**	-0.016
	(0.023)	(0.021)	(0.055)	(0.063)	(0.118)
American Indian	0.320**	0.175**	0.311**	0.110**	0.273**
	(0.025)	(0.021)	(0.025)	(0.040)	(0.065)
Chinese	-0.272**	-0.233**	-0.401**	-0.360**	-0.591**
	(0.020)	(0.015)	(0.091)	(0.045)	(0.141)
Asian Indian	-0.189**	-0.179**	-0.319**	-0.240**	-0.516**
	(0.026)	(0.023)	(0.095)	(0.048)	(0.136)
Korean	-0.212**	-0.160**	-0.384**	-0.296**	-0.564**
	(0.024)	(0.014)	(0.098)	(0.049)	(0.140)
Japanese	-0.200**	-0.128**	-0.272**	-0.226**	-0.366**
	(0.019)	(0.015)	(0.060)	(0.042)	(0.086)
Filipino	-0.018	-0.090**	-0.252**	-0.228**	-0.457**
	(0.019)	(0.011)	(0.097)	(0.048)	(0.137)
Vietnamese	-0.145**	-0.199**	-0.355**	-0.233**	-0.567**
	(0.029)	(0.022)	(0.125)	(0.037)	(0.173)
Constant	2.335**	-0.609	-1.909	-1.308	-1.737
	(0.089)	(3.186)	(3.138)	(3.088)	(3.202)
Observations	1,524	1,524	1,524	1,524	1,524
R-squared	0.477	0.813	0.827	0.824	0.828
Race/Ethnic & Year Indicators	Y	Y	Y	Y	Y
Controls for Parental Characteristics		Y	Y	Y	Y
Controls for Cultural Measures			Y		Y
Controls for Economic Measures				Y	Y

NVSS 1969-2018, limited to births at parities 1-3 (live birth orders 1-3).

All models include indicators for each race/ethnic category and year. Full model includes controls for mean maternal and paternal age, marital status, proportion of mothers who live in the U.S., and aggregate group characteristics from Census and ACS data measured separately by gender: proportions not in the labor force, foreign born, living in a three-generation household, non-U.S. citizen, home ownership, Hispanic ethnicity, farm residence, and mean values of years living in the U.S., family income, and home values.

Bootstrapped standard errors stratified by race/ethnicity in parentheses. ** p<0.01, * p<0.05, + p<0.1

Table A5: Predicted Mean Live Birth Order by Maternal Race/Ethnicity and Education

	(1)	(2)	(3)	(4)	(5)
VARIABLES			Mean Live Birth Order		
African American	0.191**	-0.013	0.193**	-0.208**	0.075
	(0.025)	(0.024)	(0.053)	(0.063)	(0.113)
American Indian	0.355**	0.232**	0.369**	0.173**	0.327**
	(0.026)	(0.024)	(0.026)	(0.039)	(0.064)
Chinese	-0.279**	-0.214**	-0.315**	-0.339**	-0.512**
	(0.022)	(0.023)	(0.091)	(0.047)	(0.145)
Asian Indian	-0.130**	-0.095**	-0.172+	-0.167**	-0.387**
	(0.028)	(0.030)	(0.097)	(0.051)	(0.143)
Korean	-0.211**	-0.180**	-0.328**	-0.309**	-0.518**
	(0.032)	(0.024)	(0.099)	(0.055)	(0.144)
Japanese	-0.207**	-0.143**	-0.247**	-0.231**	-0.341**
	(0.024)	(0.025)	(0.062)	(0.045)	(0.084)
Filipino	-0.022	-0.104**	-0.191*	-0.233**	-0.402**
	(0.023)	(0.018)	(0.097)	(0.049)	(0.143)
Vietnamese	-0.132**	-0.166**	-0.233+	-0.202**	-0.462*
	(0.034)	(0.036)	(0.125)	(0.043)	(0.180)
Mother Has Any College Education	-0.276**	-0.055**	-0.048**	-0.052**	-0.048**
	(0.018)	(0.018)	(0.018)	(0.018)	(0.017)
African American * Mom Any College	-0.116**	-0.098**	-0.097**	-0.097**	-0.098**
	(0.030)	(0.022)	(0.022)	(0.022)	(0.022)
American Indian * Mom Any College	-0.071*	-0.063*	-0.063*	-0.063*	-0.064*
	(0.036)	(0.028)	(0.028)	(0.028)	(0.028)
Chinese * Mom Any College	0.015	-0.064**	-0.062**	-0.064**	-0.062**
	(0.024)	(0.021)	(0.021)	(0.021)	(0.020)
Asian Indian * Mom Any College	-0.118**	-0.172**	-0.169**	-0.173**	-0.169**
	(0.033)	(0.025)	(0.026)	(0.025)	(0.026)
Korean * Mom Any College	-0.002	0.012	0.016	0.012	0.015
	(0.033)	(0.024)	(0.025)	(0.025)	(0.025)
Japanese * Mom Any College	0.013	-0.009	-0.010	-0.009	-0.010
	(0.025)	(0.025)	(0.025)	(0.025)	(0.025)
Filipino * Mom Any College	0.007	0.047*	0.053**	0.049**	0.053**
	(0.024)	(0.019)	(0.019)	(0.018)	(0.018)
Vietnamese * Mom Any College	-0.027	-0.056+	-0.051+	-0.057*	-0.051+
	(0.035)	(0.032)	(0.028)	(0.028)	(0.027)
Constant	2.472**	0.456	-1.108	-0.431	-0.992
	(0.073)	(2.818)	(2.782)	(2.704)	(2.826)
Observations	1,524	1,524	1,524	1,524	1,524
R-squared	0.752	0.835	0.846	0.844	0.847
Race/Ethnic, Educ, & Year Indicators	Y	Y	Y	Y	Y
Controls for Parental Characteristics		Y	Y	Y	Y
Controls for Cultural Measures			Y		Y
Controls for Economic Measures				Y	Y

NVSS 1969-2018, limited to births at third parity (live birth order 3).

All models include indicators for each race/ethnic category, maternal college education, and year. Full model includes controls for mean maternal and paternal age, marital status, proportion of mothers who live in the U.S., and aggregate group characteristics from Census and ACS data measured separately by gender: proportions not in the labor force, foreign born, living in a three-generation household, non-U.S. citizen, home ownership, Hispanic ethnicity, farm residence, and mean values of years living in the U.S., family income, and home values.

Bootstrapped standard errors stratified by race/ethnicity in parentheses. ** p<0.01, * p<0.05, + p<0.1

Table A6: Predicted Mean Live Birth Order by Maternal Race/Ethnicity and Year

	(1)	(2)	(3)	(4)	(5)
VARIABLES	Mean Live Birth Order				
African American	3.320	-0.628	-1.643	2.275	2.234
	(4.357)	(2.166)	(3.812)	(3.236)	(4.914)
American Indian	4.747	0.266	5.501*	3.295	3.663
	(4.792)	(3.458)	(2.421)	(2.568)	(3.696)
Chinese	12.738**	5.293**	9.297*	11.562**	17.820**
	(3.352)	(1.917)	(3.699)	(3.368)	(5.147)
Asian Indian	8.354	-4.994	-6.946	-3.392	-6.494
	(5.483)	(3.374)	(4.264)	(4.702)	(6.505)
Korean	-1.396	-6.862*	-4.943	-0.981	4.442
	(6.073)	(3.006)	(7.271)	(5.081)	(8.662)
Japanese	5.827+	-4.466*	0.075	-1.485	10.084
	(3.218)	(1.904)	(5.934)	(2.967)	(6.296)
Filipino	10.849**	7.807**	8.645	12.044**	20.480**
	(3.204)	(1.853)	(5.290)	(3.297)	(5.262)
Vietnamese	18.035*	19.189**	18.183**	31.976**	31.598**
	(7.277)	(3.957)	(5.996)	(5.097)	(8.105)
Year	-0.001	-0.004**	-0.002	-0.004	0.004
	(0.002)	(0.001)	(0.003)	(0.003)	(0.003)
African American * Year	-0.002	0.000	0.001	-0.001	-0.001
	(0.002)	(0.001)	(0.002)	(0.002)	(0.002)
American Indian * Year	-0.002	-0.000	-0.003*	-0.002	-0.002
	(0.002)	(0.002)	(0.001)	(0.001)	(0.002)
Chinese * Year	-0.007**	-0.003**	-0.005*	-0.006**	-0.009**
	(0.002)	(0.001)	(0.002)	(0.002)	(0.003)
Asian Indian * Year	-0.004	0.002	0.003	0.002	0.003
	(0.003)	(0.002)	(0.002)	(0.002)	(0.003)
Korean * Year	0.001	0.003*	0.002	0.000	-0.002
	(0.003)	(0.002)	(0.004)	(0.003)	(0.004)
Japanese * Year	-0.003+	0.002*	-0.000	0.001	-0.005
	(0.002)	(0.001)	(0.003)	(0.001)	(0.003)
Filipino * Year	-0.005**	-0.004**	-0.004+	-0.006**	-0.010**
	(0.002)	(0.001)	(0.003)	(0.002)	(0.003)
Vietnamese * Year	-0.009*	-0.010**	-0.009**	-0.016**	-0.016**
	(0.004)	(0.002)	(0.003)	(0.003)	(0.004)
Constant	4.026	6.351	2.356	5.914	-11.207
	(4.066)	(4.612)	(6.380)	(6.536)	(7.712)
Observations	1,524	1,524	1,524	1,524	1,524
R-squared	0.489	0.826	0.833	0.831	0.834
Race/Ethnic & Year Indicators	Y	Y	Y	Y	Y
Controls for Parental Characteristics		Y	Y	Y	Y
Controls for Cultural Measures			Y		Y
Controls for Economic Measures				Y	Y

NVSS 1969-2018, limited to births at third parity (live birth order 3).

All models include indicators for each race/ethnic category and year. Full model includes controls for mean maternal and paternal age, marital status, proportion of mothers who live in the U.S., and aggregate group characteristics from Census and ACS data measured separately by gender: proportions not in the labor force, foreign born, living in a three-generation household, non-U.S. citizen, home ownership, Hispanic ethnicity, farm residence, and mean values of years living in the U.S., family income, and home values.

Bootstrapped standard errors stratified by race/ethnicity in parentheses. ** $p<0.01$, * $p<0.05$, + $p<0.1$

Table A7: Predicted Male:Female Prenatal Visits by Maternal Race/Ethnicity and Birth Order

	(1)	(2)	(3)	(4)	(5)
VARIABLES	Male:Female Prenatal Visits				
African American	-0.025	-0.025	-0.035	-0.029	0.016
	(0.016)	(0.017)	(0.022)	(0.024)	(0.044)
American Indian	-0.013	-0.013	-0.025+	-0.033	-0.009
	(0.012)	(0.013)	(0.015)	(0.026)	(0.023)
Chinese	-0.014	-0.010	-0.043	0.015	0.064
	(0.012)	(0.013)	(0.057)	(0.015)	(0.054)
Asian Indian	-0.014	-0.008	-0.047	0.019	0.048
	(0.012)	(0.014)	(0.061)	(0.018)	(0.065)
Korean	-0.015	-0.011	-0.050	-0.000	0.056
	(0.013)	(0.013)	(0.063)	(0.013)	(0.050)
Japanese	-0.016	-0.015	-0.036	-0.002	0.013
	(0.012)	(0.013)	(0.029)	(0.015)	(0.028)
Filipino	-0.020	-0.017	-0.048	0.007	0.062
	(0.012)	(0.013)	(0.056)	(0.016)	(0.051)
Vietnamese	-0.017	-0.009	-0.060	-0.003	0.069
	(0.012)	(0.014)	(0.076)	(0.014)	(0.059)
Birth Order 2	-0.008	-0.007	-0.006	-0.007	-0.010
	(0.017)	(0.017)	(0.017)	(0.017)	(0.018)
Birth Order 3	-0.023	-0.021	-0.019	-0.020	-0.025
	(0.015)	(0.015)	(0.015)	(0.015)	(0.015)
African American – Birth Order 2	0.028	0.029	0.028	0.028	0.028
	(0.021)	(0.021)	(0.021)	(0.021)	(0.021)
African American – Birth Order 3	0.027	0.028	0.028	0.028	0.028
	(0.018)	(0.017)	(0.017)	(0.017)	(0.017)
American Indian – Birth Order 2	0.009	0.009	0.009	0.009	0.009
	(0.017)	(0.017)	(0.017)	(0.017)	(0.017)
American Indian – Birth Order 3	0.016	0.016	0.016	0.016	0.016
	(0.015)	(0.015)	(0.015)	(0.015)	(0.015)
Chinese – Birth Order 2	0.007	0.007	0.007	0.007	0.008
	(0.017)	(0.017)	(0.017)	(0.017)	(0.017)
Chinese – Birth Order 3	0.017	0.017	0.017	0.017	0.019
	(0.015)	(0.016)	(0.016)	(0.016)	(0.016)
Asian Indian – Birth Order 2	0.008	0.008	0.009	0.008	0.010
	(0.018)	(0.017)	(0.017)	(0.017)	(0.018)
Asian Indian – Birther Order 3	0.029+	0.030+	0.031+	0.029+	0.033*
	(0.015)	(0.015)	(0.016)	(0.016)	(0.016)
Korean – Birth Order 2	0.011	0.011	0.011	0.011	0.011
	(0.019)	(0.019)	(0.019)	(0.019)	(0.019)
Korean – Birth Order 3	0.015	0.015	0.015	0.015	0.016
	(0.016)	(0.016)	(0.016)	(0.016)	(0.016)
Japanese – Birth Order 2	0.010	0.010	0.010	0.010	0.010
	(0.018)	(0.018)	(0.018)	(0.018)	(0.018)
Japanese – Birth Order 3	0.037*	0.037*	0.036*	0.037*	0.037*

	(0.017)	(0.017)	(0.017)	(0.017)	(0.017)
Filipino – Birth Order 2	0.013	0.013	0.013	0.013	0.012
	(0.018)	(0.018)	(0.018)	(0.018)	(0.018)
Filipino – Birth Order 3	0.030+	0.030+	0.029+	0.030+	0.028+
	(0.016)	(0.016)	(0.016)	(0.016)	(0.016)
Vietnamese – Birth Order 2	0.010	0.009	0.009	0.009	0.009
	(0.017)	(0.017)	(0.017)	(0.017)	(0.017)
Vietnamese – Birth Order 3	0.026+	0.025+	0.024+	0.024+	0.025+
	(0.014)	(0.014)	(0.014)	(0.014)	(0.015)
Constant	0.990**	2.235**	2.415**	2.312**	2.494**
	(0.294)	(0.802)	(0.793)	(0.773)	(0.797)
Observations	4,372	4,372	4,372	4,372	4,372
R-squared	0.008	0.009	0.015	0.012	0.017
Race/Ethnic, Birth Order, & Year Indicators	Y	Y	Y	Y	Y
Controls for Parental Characteristics		Y	Y	Y	Y
Controls for Cultural Measures			Y		Y
Controls for Economic Measures				Y	Y

NVSS 1969-2018, limited to births at parities 1-3 (live birth orders 1-3).

All models include indicators for each race/ethnic category, birth order, and year. Full model includes controls for mean maternal and paternal age, marital status, proportion of mothers who live in the U.S., proportion of births at 1st, 2nd, and 3rd parity, and aggregate group characteristics from Census and ACS data measured separately by gender: proportions not in the labor force, foreign born, living in a three-generation household, non-U.S. citizen, home ownership, Hispanic ethnicity, farm residence, and mean values of years living in the U.S., family income, and home values.

Bootstrapped standard errors stratified by race/ethnicity in parentheses. ** p<0.01, * p<0.05, + p<0.1

Table A8: Predicted Male:Female Prenatal Visits by Maternal Race/Ethnicity and Education

	(1)	(2)	(3)	(4)	(5)
VARIABLES	Male:Female Prenatal Visits				
African American	0.014*	0.035+	0.002	0.038	-0.064
	(0.007)	(0.020)	(0.038)	(0.035)	(0.076)
American Indian	-0.003	0.006	-0.014	-0.001	-0.067+
	(0.005)	(0.012)	(0.017)	(0.021)	(0.036)
Chinese	0.003	0.016	0.080	0.031	0.052
	(0.006)	(0.014)	(0.059)	(0.028)	(0.101)
Asian Indian	0.017**	0.037+	0.102+	0.057	0.126
	(0.006)	(0.020)	(0.062)	(0.036)	(0.107)
Korean	-0.013	-0.002	0.076	0.014	0.039
	(0.019)	(0.022)	(0.068)	(0.032)	(0.097)
Japanese	0.023	0.024	0.053	0.036+	0.054
	(0.015)	(0.015)	(0.035)	(0.020)	(0.049)
Filipino	0.013+	0.022*	0.080	0.041	0.029
	(0.007)	(0.009)	(0.058)	(0.028)	(0.101)
Vietnamese	0.010+	0.032+	0.100	0.032	0.040
	(0.006)	(0.017)	(0.071)	(0.024)	(0.111)
Mother Has Any College Education	0.005	0.003	0.002	0.003	0.001
	(0.004)	(0.007)	(0.007)	(0.007)	(0.007)
African American * Mom Any College	-0.017+	-0.025*	-0.025*	-0.026*	-0.025+
	(0.009)	(0.013)	(0.012)	(0.013)	(0.014)
American Indian * Mom Any College	-0.000	-0.003	-0.003	-0.003	-0.000
	(0.007)	(0.007)	(0.007)	(0.007)	(0.009)
Chinese * Mom Any College	-0.012	-0.013	-0.013	-0.013	-0.017
	(0.009)	(0.009)	(0.009)	(0.009)	(0.012)
Asian Indian * Mom Any College	-0.016*	-0.022**	-0.023**	-0.023**	-0.024
	(0.007)	(0.008)	(0.008)	(0.008)	(0.014)
Korean * Mom Any College	0.015	0.010	0.010	0.009	0.009
	(0.020)	(0.021)	(0.021)	(0.021)	(0.022)
Japanese * Mom Any College	-0.016	-0.012	-0.011	-0.012	-0.010
	(0.015)	(0.014)	(0.015)	(0.015)	(0.016)
Filipino * Mom Any College	-0.018*	-0.019*	-0.020*	-0.019*	-0.018*
	(0.008)	(0.008)	(0.008)	(0.009)	(0.008)
Vietnamese * Mom Any College	-0.014*	-0.021*	-0.021*	-0.021*	-0.022+
	(0.007)	(0.010)	(0.010)	(0.010)	(0.011)
Constant	0.385*	2.570**	2.835**	2.692*	2.930**
	(0.159)	(0.977)	(1.023)	(1.120)	(1.092)
Observations	1,456	1,456	1,456	1,456	1,456
R-squared	0.290	0.299	0.307	0.303	0.315
Race/Ethnic, Educ, & Year Indicators	Y	Y	Y	Y	Y
Controls for Parental Characteristics		Y	Y	Y	Y
Controls for Cultural Measures			Y		Y
Controls for Economic Measures				Y	Y

NVSS 1969-2018, limited to births at third parity (live birth order 3).

All models include indicators for each race/ethnic category, maternal college education, and year. Full model includes controls for mean maternal and paternal age, marital status, proportion of mothers who live in the U.S., proportion of births at 1st, 2nd, and 3rd parity, and aggregate group characteristics from Census and ACS data measured separately by gender: proportions not in the labor force, foreign born, living in a three-generation household, non-U.S. citizen, home ownership, Hispanic ethnicity, farm residence, and mean values of years living in the U.S., family income, and home values.
Bootstrapped standard errors stratified by race/ethnicity in parentheses. ** $p<0.01$, * $p<0.05$, + $p<0.1$

Table A9: Predicted Male:Female Prenatal Visits by Maternal Race/Ethnicity and Year

	(1)	(2)	(3)	(4)	(5)
VARIABLES			Male:Female Prenatal Visits		
African American	1.260	0.869	2.568*	0.969	0.734
	(0.870)	(0.865)	(1.298)	(1.436)	(2.606)
American Indian	0.033	-0.285	-0.777	-0.852	-1.969
	(0.708)	(0.715)	(1.326)	(0.754)	(2.268)
Chinese	-0.263	0.044	2.135	-0.967	-4.065
	(0.801)	(0.854)	(1.407)	(1.671)	(2.912)
Asian Indian	0.375	0.709	0.745	-0.840	-3.978
	(1.028)	(1.313)	(2.148)	(1.831)	(3.971)
Korean	-0.387	-0.613	3.974	-1.847	-3.703
	(2.042)	(1.985)	(3.013)	(3.209)	(5.117)
Japanese	0.475	0.284	7.300*	0.746	4.399
	(1.132)	(1.234)	(3.062)	(2.261)	(3.348)
Filipino	1.109+	0.660	1.272	-0.799	-4.592+
	(0.668)	(0.739)	(1.769)	(1.759)	(2.783)
Vietnamese	-2.063+	-3.375**	-5.418	-8.307**	-13.905*
	(1.076)	(1.239)	(4.058)	(3.076)	(5.925)
Year	0.013**	0.014**	0.016**	0.014**	0.012**
	(0.003)	(0.003)	(0.003)	(0.004)	(0.004)
African American * Year	-0.001	-0.000	-0.001*	-0.001	-0.000
	(0.000)	(0.000)	(0.001)	(0.001)	(0.001)
American Indian * Year	-0.000	0.000	0.000	0.000	0.001
	(0.000)	(0.000)	(0.001)	(0.000)	(0.001)
Chinese * Year	0.000	-0.000	-0.001	0.000	0.002
	(0.000)	(0.000)	(0.001)	(0.001)	(0.001)
Asian Indian * Year	-0.000	-0.000	-0.000	0.000	0.002
	(0.001)	(0.001)	(0.001)	(0.001)	(0.002)
Korean * Year	0.000	0.000	-0.002	0.001	0.002
	(0.001)	(0.001)	(0.001)	(0.002)	(0.003)
Japanese * Year	-0.000	-0.000	-0.004*	-0.000	-0.002
	(0.001)	(0.001)	(0.002)	(0.001)	(0.002)
Filipino * Year	-0.001+	-0.000	-0.001	0.000	0.002
	(0.000)	(0.000)	(0.001)	(0.001)	(0.001)
Vietnamese * Year	0.001+	0.002**	0.003	0.004**	0.007*
	(0.001)	(0.001)	(0.002)	(0.002)	(0.003)
Constant	-25.625**	-24.482**	-27.892**	-24.590**	-20.989**
	(6.833)	(7.087)	(6.815)	(7.633)	(7.798)
Observations	1,456	1,456	1,456	1,456	1,456
R-squared	0.288	0.296	0.307	0.303	0.316
Race/Ethnic & Year Indicators	Y	Y	Y	Y	Y
Controls for Parental Characteristics		Y	Y	Y	Y
Controls for Cultural Measures			Y		Y
Controls for Economic Measures				Y	Y

NVSS 1969-2018, limited to births at third parity (live birth order 3).

All models include indicators for each race/ethnic category and year. Full model includes controls for mean maternal and paternal age, marital status, proportion of mothers who live in the U.S., proportion of births at 1st, 2nd, and 3rd parity, and aggregate group characteristics from Census and ACS data measured separately by gender: proportions not in the labor force, foreign born, living in a three-generation household, non-U.S. citizen, home ownership, Hispanic ethnicity, farm residence, and mean values of years living in the U.S., family income, and home values.

Bootstrapped standard errors stratified by race/ethnicity in parentheses. ** $p<0.01$, * $p<0.05$, + $p<0.1$

Figure A1: Mean Live Birth Order and Proportion of Births by Parity and Race/Ethnicity

Panel A.

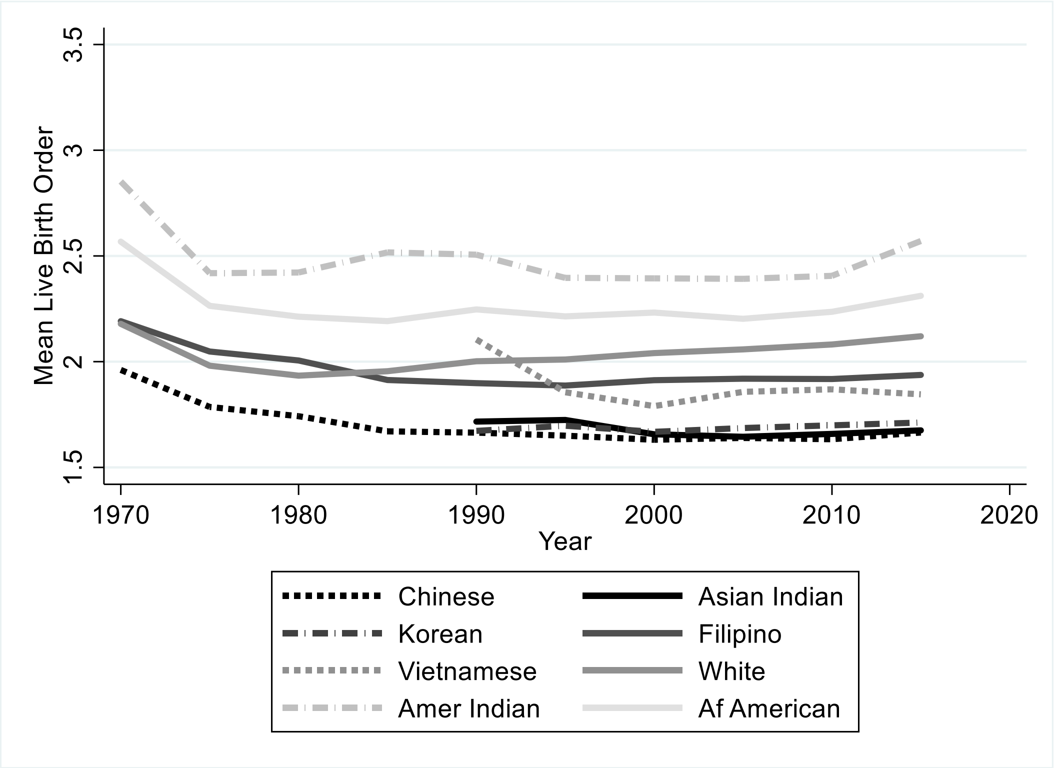

Panel B.

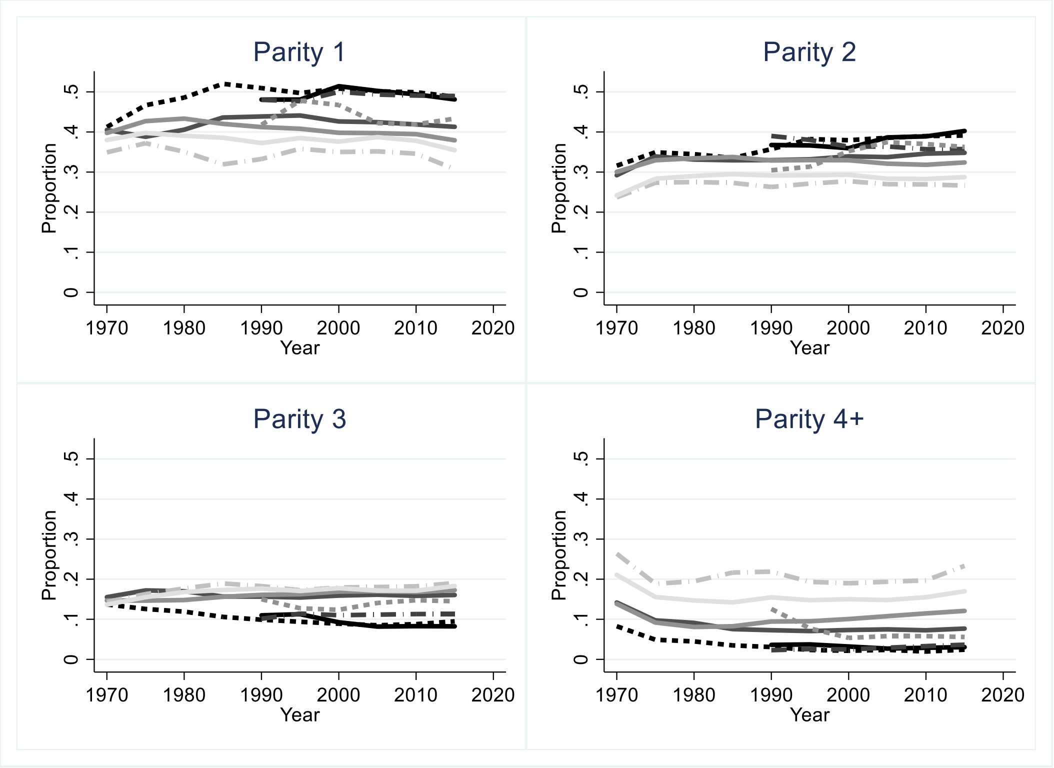

NVSS 1969-2018. Panel A: mean live births by race/ethnic group over time. Panel B: proportion of births by parity within race/ethnic group. Includes live birth orders 1-3 and 4 and above.

Figure A2: Predicted Infant Sex Ratios by Maternal Education Category and Race/Ethnicity at Parities 1-3

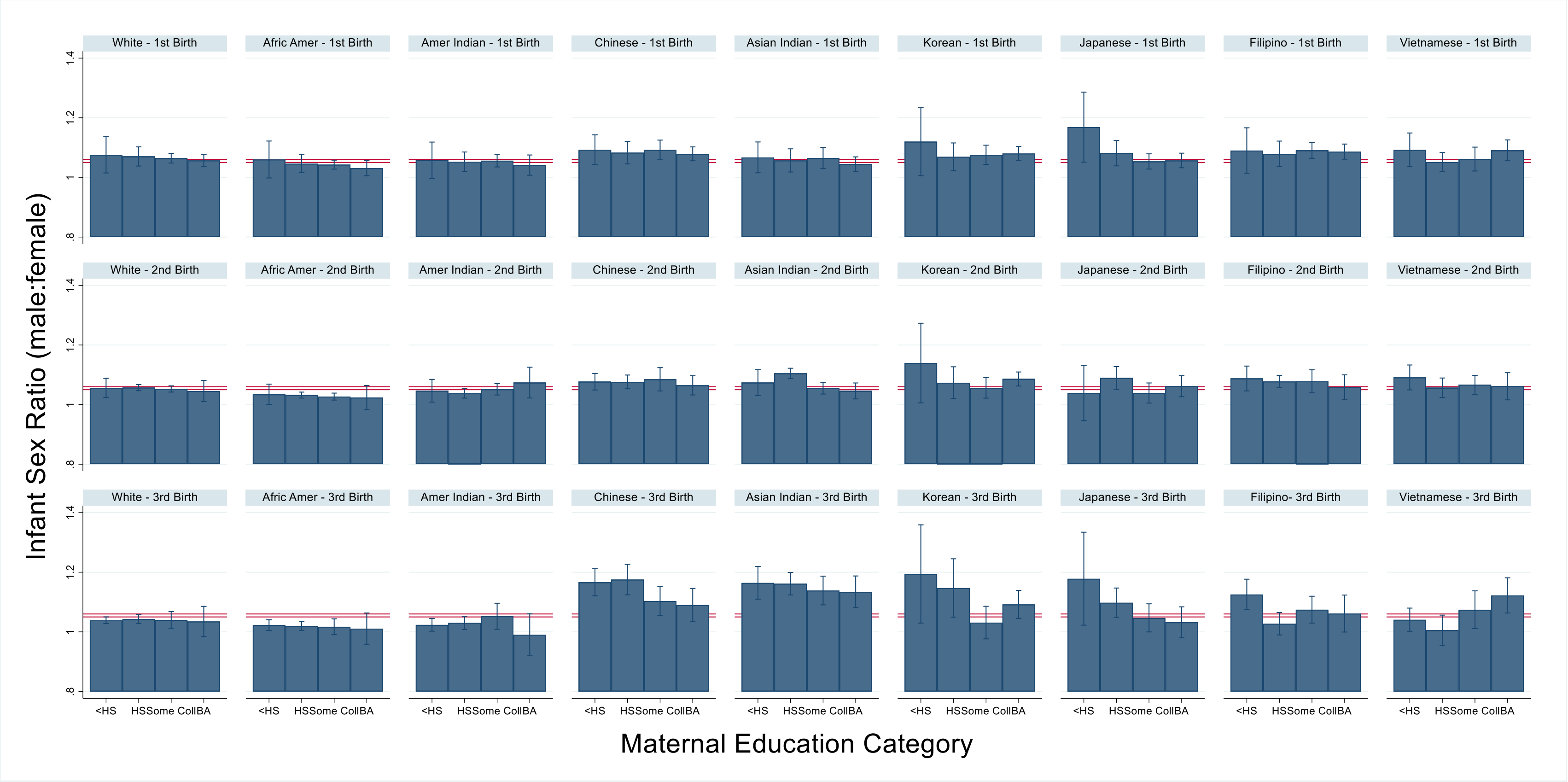

NVSS 1969-2018, limited to births at parities 1-3.

Red horizontal lines indicate infant sex ratios within the biological norm.

Model includes indicators for each race/ethnic category, maternal education category, and year, and controls for mean maternal and paternal age, marital status, and proportion of mothers who live in the U.S.

Supplementary Online Appendix: Analyses Conditional on Sex of Previous Children

Data and Methods

Using pooled American Community Survey data for years 2000-2018 from IPUMS (Ruggles et al. 2018), we follow previous research (Almond and Edlund 2008) and restrict the sample to families in which: 1) one parent is the household head; 2) all children were born in the U.S.; 3) none of the children are adopted or step-children; and 4) the eldest child is less than 13 years old to reduce the chances that an older child has left the household. We focus on the eldest three children (parity one through three) and further exclude children who are from multiple births (e.g., twins). This yields an analysis sample of 2,945,642 children in 971,150 families.

Within this sample, we assign parity based on the age of children in the household and examine child sex ratios by parity and the sex of older children. We focus particularly on 175,311 children born at third parity with either two previous girl or two previous boy children.

Parental education categories include those with less than high school, a high school degree, some college, or at least a college degree. We estimate the likelihood of having a boy at third parity among parents with no college education compared to those with at least some college education. We examine variation between parents with and without any college because it enhances income potential (Averett and Burton 1996; Giani et al. 2020) and exposes people to more cosmopolitan cultural beliefs (Baker 2014; Greenwood 1975). We examine variation by maternal education and we also compare by paternal education because male attitudes, preferences, and earning potential hold more cultural value in traditional or patriarchal cultures (e.g., China, South Korea, and India; Jha et al. 2006; Yi et al. 1993; Chung and Gupta 2007; Gietel-Basten et al. 2018). In sensitivity analyses, we find similar results when using paternal identification (rather than maternal) to define race/ethnicity category and find similar results.

For each third child with either two older brothers or two older sisters, we estimate the likelihood of being a boy. Out of concern about models that use non-linear link functions (Mood 2010; Gomila 2020), we use linear probability models to predict likelihood of a boy child at third parity. Equation A1 predicts the likelihood that a third child (i) is a boy, with the sex mix of previous children, and indicators for each maternal race/ethnic category (j), education category (k), and year (t). Robust standard errors are adjusted for clustering within maternal race/ethnic category. The coefficients of interest (β_j) test whether the likelihood a third child with two sisters is a boy differs by race/ethnic category. For each race/ethnic group, these coefficients compare the likelihood of having a boy at third parity when the two previous children are girls rather than boys. These analyses allow a further test of *hypotheses 1a and 1b* in the main analyses, conditional on the sex of previous children.

$$\text{Boy}_{ijkt} = \beta_j \text{Sisters}_{ijkt} * \text{Race/Ethnic}_j + \text{Educ}_k + \text{Year}_t + W_{ijkt} + \varepsilon_{ijkt} \quad \text{(A1)}$$

To test the other hypotheses, we limit the sample to third children with two older sisters. Using this sample, coefficients for maternal education categories (β_k) interacted with each race/ethnic category test whether the likelihood of having a boy after two previous girls differs significantly by maternal education (*hypothesis 2*). Coefficients for a continuous year measure interacted with race/ethnic category test whether trends differ in likelihood of having a boy after two previous girls (*hypothesis 3*). To test *hypothesis 4*, we stratify the sample by paternal college education and repeat analyses for *hypothesis 2*, with interaction terms for maternal education and race/ethnic category. We follow the same approach as the main analyses to test whether

coefficients for maternal college education by race/ethnicity differ significantly among children of fathers with and without college education (Paternoster et al. 1998; Clogg et al. 1995).

We fit these models with and without controls for parental and family characteristics (*W*). Parental characteristics include maternal and paternal age and marital status. Economic measures include separate indicators for whether the mother or father is not in the labor force, indicators for home ownership and residence on a farm, home value (logged to reduce skewness), and family and household income (logged). Cultural measures include separate indicators for whether the mother or father is foreign born or a non-citizen, separate measures for years the mother and father have resided in the U.S. (among those born outside the country), and an indicator for living in a three-generation household (e.g., grandparents, parents, and children). The indicator for a mother who is not in the labor force is included in the economic measures; we also include it in the cultural measures because it provides a proxy for traditional gender roles.

We fit models with and without these controls because previous work suggests that child sex ratios may depend on these economic and cultural characteristics (Guilmoto 2009; Bongaarts 2013). An association between parental education and child sex could be confounded by economic or cultural measures without holding them constant.

Results

Table B2 shows predicted likelihood of having a male third child by maternal race/ethnicity and sex of the two previous children. Figure B1 shows predictive margins based on the full model in Table B2. Among white mothers, third children with two previous girls are slightly, but significantly less likely to be male. This is consistent with existing evidence of a tendency to repeat the sex of earlier children (Almond and Edlund 2008; Renkonen et al. 1962). However, estimates vary by maternal race/ethnicity and suggest a significantly higher likelihood of having a boy among Chinese, Asian Indian, Korean, Japanese, and Filipino mothers when the two previous children are girls. Figure B1 shows that the difference is particularly pronounced among children of Chinese, Asian Indian, and Japanese mothers, who are respectively 4.6%, 12.7%, and 4.8% more likely to be male, when they have two older sisters compared to children of white mothers. Consistent with *hypothesis 1*, these estimates suggest male-biased child sex ratios among Chinese, Asian Indian, and Japanese families, when the two previous children are girls.

If parents from these groups are using sex selective behaviors to increase the likelihood of having a boy, then there should be a longer gap between the second and third children for those groups. Results support this; Table B3 shows estimates predicting the age gap between second and third children when the two previous children are girls. Compared to children of white mothers, Figure B2 shows there is a significantly longer predicted gap between second and third children among Chinese, Asian Indian, and Vietnamese mothers when the two previous children are girls. Among Chinese and Asian Indian mothers, this amounts to approximately 2-3 months longer between children.

Table B4 examines trends in the likelihood of having a boy when the two previous children are girls. There is no trend among whites and for most groups there is a slight increase over time in the likelihood of a male third child after two previous girls. However, among Asian Indian and Filipino mothers, there is a slight, but significant decline of about 0.5% per year since 2000. This offers some support for *hypothesis 3* regarding gender inequality, but only suggests increasing equality among Asian Indian and Filipino parents.

Table B5 shows results of models testing for variation by maternal education. For nearly every group, third children with two older sisters are significantly less likely to be boys when the mother has attended college. In the full model, these estimates range from 1% lower likelihood of a boy among Chinese to 14% among Japanese when the mother has more education. These results support *hypothesis 2*. The exceptions are third children of Asian Indian and African American mothers, who are more likely to be a boy when the mother has attended college (4% and 3%, respectively). This could reflect less exposure to environmental toxins among those with higher education, which is related to lower child sex ratios (Sakamoto et al. 2001; Figa-Talamanca and Petrelli 2000; Whorton et al. 1994; Goldsmith et al. 1984).

Table B6 compares the relationship between maternal education and likelihood of a male third child by paternal education. Coefficients for maternal college education differ significantly between models by paternal education for all race/ethnic groups (Paternoster et al. 1998). In the full model, maternal college education is related to lower likelihood of having a boy when the father has also attended college for all groups except African Americans and Vietnamese. Estimates suggest maternal education is related to a higher likelihood of having a boy for several groups when the father has not attended college. Specifically, compared to white mothers, Chinese and Asian Indian mothers are over 10% more likely to have a boy after two girls when they have attended college and the father has not. However, this relationship reverses when both parents have attended college and Chinese and Asian Indian mothers are about 10% less likely to have a boy after two girls. Results for Korean mothers also suggest maternal college education is related to more equal infant sex ratios when both parents have attended college. The positive relationship between parental college education and likelihood of having a male third child among African Americans and Vietnamese are consistent with greater equality of child sex ratios because of the female-biased ratios among African Americans (see Figure 1) and the significantly lower likelihood of having a male third child among Vietnamese (see Table B2). Overall, results support *hypothesis 4* and suggest greater equality of child sex ratios with maternal education when parents are educationally homogamous.

Results are similar if we include families with likely multiple births (i.e. children of equal age and parity). Results are also consistent when excluding families in which the mother or father is not a U.S. citizen.

Overall, results examining child sex ratios conditional on the sex of previous children are consistent with the main analyses. Results do not support the argument that education increases son-biased sex ratios by increasing access to sex-selective technologies. Rather, results indicate that education is related to less male-biased preferences among Asian parents. Education is more strongly related to equality of child sex ratios when both parents have attended college, which is consistent with the suggestion that higher education promotes more egalitarian views.

Appendix Tables

Table B1: Descriptive Statistics

Variable	Mean	Std Dev	Mom No College	Mom Any College
Boy	0.51	0.50	0.51	0.51
Age Gap between 2nd and 3rd Child	2.98	1.61	2.99	2.98
White	0.84	0.36	0.82	0.85
African American	0.10	0.30	0.13	0.08
American Indian	0.01	0.10	0.02	0.01
Chinese	0.01	0.11	0.01	0.02
Asian Indian	0.01	0.11	0.00	0.02
Korean	0.00	0.07	0.00	0.01
Japanese	0.00	0.05	0.00	0.00
Filipino	0.01	0.09	0.00	0.01
Vietnamese	0.01	0.08	0.01	0.01
1st Child	0.53	0.50	0.53	0.53
2nd Child - Previous Boy	0.19	0.39	0.18	0.19
2nd Child - Previous Girl	0.18	0.38	0.17	0.18
3rd Child - 2 Previous Boys	0.03	0.17	0.03	0.03
3rd Child - 1 Previous Boy & Girl	0.05	0.22	0.06	0.05
3rd Child - 2 Previous Girls	0.03	0.17	0.03	0.03
Maternal Educ <HS	0.06	0.24	0.19	-
Maternal Educ HS	0.27	0.45	0.81	-
Maternal Educ Some Coll	0.26	0.44	-	0.39
Maternal Educ BA	0.40	0.49	-	0.61
Year	2010.06	4.86	2009.45	2010.37
Maternal Age	35.18	6.32	32.71	36.43
Paternal Age	37.87	6.86	35.90	38.75
Married	0.79	0.40	0.68	0.85
Not in Labor Force - Mom	0.31	0.46	0.39	0.27
Not in Labor Force - Dad	0.04	0.20	0.06	0.03
Foreign Born - Mom	0.14	0.35	0.17	0.12
Foreign Born - Dad	0.15	0.36	0.21	0.13
3 Generation Household	0.03	0.18	0.04	0.03
Years in the US - Mom	32.59	9.12	29.51	34.15
Years in the US - Dad	34.96	9.81	32.02	36.26
Non-Citizen - Mom	0.06	0.24	0.11	0.04
Non-Citizen - Dad	0.07	0.25	0.12	0.04
Home Ownership	0.71	0.45	0.53	0.80
Home Value	248674.20	363938.00	117262.60	315588.00
Hispanic - Mom	0.11	0.31	0.18	0.07

Hispanic - Dad	0.11	0.31	0.20	0.07
Family Income	101765.40	105391.30	55000.14	125578.00
Farm Residence	0.01	0.11	0.01	0.01
N	2,945,642		993,842	1,951,800

ACS 2000-2018, limited to children 1-3 in households where: one parent is the household head; all children were born in the U.S.; no children are adopted or step-children; the eldest child is less than 13 years old; and children are not from multiple births.

Table B2: Predicted Likelihood of Male Third Child by Sibling Sex and Maternal Race/Ethnicity

	(1)	(2)	(3)	(4)	(5)
VARIABLES			Boy		
African American	-0.013**	-0.010**	-0.008**	-0.011**	-0.009**
	(0.000)	(0.000)	(0.001)	(0.000)	(0.001)
American Indian	-0.003**	-0.011**	-0.011**	-0.011**	-0.011**
	(0.000)	(0.001)	(0.000)	(0.000)	(0.000)
Chinese	-0.010**	-0.004**	0.000	-0.006**	-0.003
	(0.000)	(0.000)	(0.002)	(0.000)	(0.003)
Asian Indian	-0.049**	-0.052**	-0.046**	-0.053**	-0.049**
	(0.000)	(0.001)	(0.003)	(0.001)	(0.004)
Korean	-0.009**	-0.004**	-0.001	-0.005**	-0.003+
	(0.000)	(0.000)	(0.001)	(0.000)	(0.002)
Japanese	-0.016**	-0.017**	-0.017**	-0.018**	-0.018**
	(0.000)	(0.001)	(0.001)	(0.000)	(0.001)
Filipino	-0.006**	-0.004**	-0.001	-0.005**	-0.003
	(0.000)	(0.000)	(0.002)	(0.000)	(0.002)
Vietnamese	0.011**	0.010**	0.015**	0.008**	0.013**
	(0.000)	(0.001)	(0.003)	(0.000)	(0.003)
Two Previous Girls	-0.008**	-0.007**	-0.007**	-0.007**	-0.007**
	(0.000)	(0.000)	(0.000)	(0.000)	(0.000)
African American * Two Previous Girls	0.004**	0.007**	0.006**	0.007**	0.007**
	(0.000)	(0.000)	(0.000)	(0.000)	(0.000)
American Indian * Two Previous Girls	0.006**	0.010**	0.010**	0.010**	0.010**
	(0.000)	(0.000)	(0.000)	(0.000)	(0.000)
Chinese * Two Previous Girls	0.045**	0.044**	0.045**	0.045**	0.046**
	(0.000)	(0.000)	(0.000)	(0.000)	(0.000)
Asian Indian * Two Previous Girls	0.119**	0.127**	0.128**	0.126**	0.127**
	(0.000)	(0.000)	(0.000)	(0.000)	(0.000)
Korean * Two Previous Girls	0.024**	0.024**	0.024**	0.024**	0.024**
	(0.000)	(0.000)	(0.000)	(0.000)	(0.000)
Japanese * Two Previous Girls	0.042**	0.047**	0.047**	0.047**	0.048**
	(0.000)	(0.000)	(0.001)	(0.000)	(0.001)
Filipino * Two Previous Girls	0.013**	0.018**	0.018**	0.019**	0.019**
	(0.000)	(0.000)	(0.000)	(0.000)	(0.000)
Vietnamese * Two Previous Girls	0.000*	-0.005**	-0.005**	-0.005**	-0.006**
	(0.000)	(0.000)	(0.000)	(0.000)	(0.000)
Constant	0.515**	0.521**	0.521**	0.521**	0.519**
	(0.005)	(0.007)	(0.007)	(0.006)	(0.007)
Observations	175,311	151,749	151,749	151,675	151,675
R-squared	0.000	0.000	0.000	0.000	0.000
Race/Ethnic, Educ, & Year Indicators	Y	Y	Y	Y	Y
Controls for Parental Characteristics		Y	Y	Y	Y
Controls for Cultural Measures			Y		Y
Controls for Economic Measures				Y	Y

ACS 2000-2018, limited to third children with two older brothers or sisters in households where: one parent is the household head; all children were born in the U.S.; no children are adopted or step-children; the eldest child is less than 13 years old; and children are not from multiple births.

All models include indicators for each race/ethnic category, maternal education category, and year. Full model includes controls for parental characteristics :maternal and paternal age, parental marital status; economic characteristics: separate indicators for whether the mother or father is not in the labor force, indicators for home ownership and residence on a farm, home value (logged), and family and household income (logged); and cultural measures: indicator for whether mother is not in labor force, separate indicators for whether the mother or father is foreign born or a non-citizen, separate measures for years the mother and father have resided in the U.S., indicator for living in a three-generation household (e.g., grandparents, parents, and children).

Robust standard errors adjusted for race/ethnic clustering in parentheses. ** $p<0.01$, * $p<0.05$, + $p<0.1$

Table B3: Predicted Age Gap between Second and Third Child with Two Previous Girls by Maternal Race/Ethnicity

	(1)	(2)	(3)	(4)	(5)
VARIABLES	Age Gap between Second and Third Child (years)				
African American	-0.054**	-0.009	-0.035*	0.027**	0.022
	(0.008)	(0.007)	(0.012)	(0.007)	(0.014)
American Indian	-0.045**	0.005	0.014	0.021	0.027*
	(0.011)	(0.014)	(0.010)	(0.012)	(0.009)
Chinese	0.226**	0.119**	0.031	0.160**	0.136**
	(0.001)	(0.001)	(0.034)	(0.003)	(0.036)
Asian Indian	0.332**	0.275**	0.186**	0.317**	0.286**
	(0.006)	(0.002)	(0.040)	(0.002)	(0.041)
Korean	0.070**	-0.006	-0.092**	0.049**	0.016
	(0.008)	(0.006)	(0.024)	(0.003)	(0.029)
Japanese	0.118**	0.012	-0.021*	0.044**	0.040**
	(0.010)	(0.009)	(0.007)	(0.006)	(0.009)
Filipino	-0.004	-0.057**	-0.132**	-0.037**	-0.046
	(0.007)	(0.004)	(0.026)	(0.002)	(0.028)
Vietnamese	0.178**	0.078**	-0.025	0.114**	0.079
	(0.003)	(0.006)	(0.043)	(0.006)	(0.045)
Constant	2.946**	2.143**	2.130**	1.902**	1.879**
	(0.030)	(0.034)	(0.023)	(0.028)	(0.031)
Observations	83,061	71,649	71,649	71,604	71,604
R-squared	0.002	0.009	0.013	0.015	0.016
Race/Ethnic, Educ, & Year Indicators	Y	Y	Y	Y	Y
Controls for Parental Characteristics		Y	Y	Y	Y
Controls for Cultural Measures			Y		Y
Controls for Economic Measures				Y	Y

ACS 2000-2018, limited to third children with two older sisters in households where: one parent is the household head; all children were born in the U.S.; no children are adopted or step-children; the eldest child is less than 13 years old; and children are not from multiple births.

All models include indicators for each race/ethnic category, maternal education category, and year. Full model includes controls for parental characteristics :maternal and paternal age, parental marital status; economic characteristics: separate indicators for whether the mother or father is not in the labor force, indicators for home ownership and residence on a farm, home value (logged), and family and household income (logged); and cultural measures: indicator for whether mother is not in labor force, separate indicators for whether the mother or father is foreign born or a non-citizen, separate measures for years the mother and father have resided in the U.S., indicator for living in a three-generation household (e.g., grandparents, parents, and children).

Robust standard errors adjusted for race/ethnic clustering in parentheses. ** p<0.01, * p<0.05, + p<0.1

Table B4: Predicted Likelihood of Male Third Child with Two Previous Girls by Maternal Race/Ethnicity and Year

	(1)	(2)	(3)	(4)	(5)
VARIABLES			Boy		
African American	-4.780**	-3.896**	-4.243**	-3.751**	-4.072**
	(0.069)	(0.032)	(0.103)	(0.037)	(0.104)
American Indian	0.515**	-0.896**	-0.866**	-0.970**	-0.983**
	(0.043)	(0.084)	(0.079)	(0.084)	(0.087)
Chinese	-4.979**	-3.200**	-3.212**	-3.078**	-3.132**
	(0.043)	(0.055)	(0.189)	(0.100)	(0.155)
Asian Indian	7.483**	9.215**	9.805**	9.319**	9.817**
	(0.171)	(0.081)	(0.258)	(0.074)	(0.177)
Korean	-5.339**	-3.715**	-3.462**	-3.527**	-3.218**
	(0.113)	(0.071)	(0.219)	(0.079)	(0.211)
Japanese	-19.283**	-21.748**	-21.268**	-21.708**	-21.375**
	(0.115)	(0.112)	(0.248)	(0.164)	(0.288)
Filipino	3.833**	8.406**	8.510**	8.462**	8.434**
	(0.090)	(0.045)	(0.061)	(0.058)	(0.080)
Vietnamese	-15.354**	-19.836**	-19.708**	-19.879**	-19.853**
	(0.265)	(0.099)	(0.156)	(0.091)	(0.114)
Year	-0.000	-0.000	-0.000	-0.000	-0.000
	(0.000)	(0.000)	(0.000)	(0.000)	(0.000)
African American * Year	0.002**	0.002**	0.002**	0.002**	0.002**
	(0.000)	(0.000)	(0.000)	(0.000)	(0.000)
American Indian * Year	-0.000**	0.000**	0.000**	0.000**	0.000**
	(0.000)	(0.000)	(0.000)	(0.000)	(0.000)
Chinese * Year	0.002**	0.002**	0.002**	0.002**	0.002**
	(0.000)	(0.000)	(0.000)	(0.000)	(0.000)
Asian Indian * Year	-0.004**	-0.005**	-0.005**	-0.005**	-0.005**
	(0.000)	(0.000)	(0.000)	(0.000)	(0.000)
Korean * Year	0.003**	0.002**	0.002**	0.002**	0.002**
	(0.000)	(0.000)	(0.000)	(0.000)	(0.000)
Japanese * Year	0.010**	0.011**	0.011**	0.011**	0.011**
	(0.000)	(0.000)	(0.000)	(0.000)	(0.000)
Filipino * Year	-0.002**	-0.004**	-0.004**	-0.004**	-0.004**
	(0.000)	(0.000)	(0.000)	(0.000)	(0.000)
Vietnamese * Year	0.008**	0.010**	0.010**	0.010**	0.010**
	(0.000)	(0.000)	(0.000)	(0.000)	(0.000)
Constant	0.929	1.227	1.210	1.257	1.273
	(0.557)	(0.876)	(0.874)	(0.867)	(0.869)
Observations	83,061	71,649	71,649	71,604	71,604
R-squared	0.000	0.001	0.001	0.001	0.001
Race/Ethnic, Educ, & Year FE	Y	Y	Y	Y	Y
Controls for Parental Characteristics		Y	Y	Y	Y
Controls for Cultural Measures			Y		Y
Controls for Economic Measures				Y	Y

ACS 2000-2018, limited to third children with two older sisters in households where: one parent is the household head; all children were born in the U.S.; no children are adopted or step-children; the eldest child is less than 13 years old; and children are not from multiple births.

All models include indicators for each race/ethnic category, maternal education category, and year. Full model includes controls for parental characteristics :maternal and paternal age, parental marital status; economic characteristics: separate indicators for whether the mother or father is not in the labor force, indicators for home ownership and residence on a farm, home value (logged), and family and household income (logged); and cultural measures: indicator for whether mother is not in labor force, separate indicators for whether the mother or father is foreign born or a non-citizen, separate measures for years the mother and father have resided in the U.S., indicator for living in a three-generation household (e.g., grandparents, parents, and children).

Robust standard errors adjusted for race/ethnic clustering in parentheses. ** p<0.01, * p<0.05, + p<0.1

Table B5: Predicted Likelihood of Male Third Child by Maternal Race/Ethnicity and Education

VARIABLES	(1)	(2)	(3)<br>Boy	(4)	(5)
African American	-0.016**	-0.022**	-0.022**	-0.027**	-0.025**
	(0.000)	(0.001)	(0.001)	(0.001)	(0.001)
American Indian	0.019**	0.020**	0.019**	0.018**	0.018**
	(0.000)	(0.002)	(0.002)	(0.001)	(0.001)
Chinese	0.027**	0.045**	0.057**	0.040**	0.051**
	(0.001)	(0.001)	(0.004)	(0.001)	(0.004)
Asian Indian	0.061**	0.053**	0.068**	0.050**	0.063**
	(0.001)	(0.001)	(0.004)	(0.001)	(0.004)
Korean	0.028**	0.040**	0.050**	0.037**	0.045**
	(0.001)	(0.002)	(0.002)	(0.001)	(0.002)
Japanese	0.181**	0.160**	0.162**	0.158**	0.159**
	(0.001)	(0.001)	(0.001)	(0.001)	(0.002)
Filipino	0.031**	0.044**	0.052**	0.042**	0.049**
	(0.001)	(0.001)	(0.002)	(0.001)	(0.002)
Vietnamese	0.027**	0.016**	0.028**	0.013**	0.023**
	(0.000)	(0.001)	(0.005)	(0.001)	(0.004)
Mother Has Any College Education	0.004	0.000	-0.006*	-0.006**	-0.008*
	(0.003)	(0.001)	(0.002)	(0.002)	(0.003)
African American * Mom Any College	0.018**	0.033**	0.035**	0.036**	0.037**
	(0.001)	(0.001)	(0.001)	(0.001)	(0.001)
American Indian * Mom Any College	-0.039**	-0.052**	-0.051**	-0.050**	-0.049**
	(0.001)	(0.001)	(0.001)	(0.001)	(0.001)
Chinese * Mom Any College	0.012**	-0.007**	-0.008**	-0.003**	-0.006**
	(0.001)	(0.001)	(0.001)	(0.001)	(0.001)
Asian Indian * Mom Any College	0.012**	0.029**	0.028**	0.030**	0.028**
	(0.001)	(0.001)	(0.000)	(0.001)	(0.000)
Korean * Mom Any College	-0.016**	-0.025**	-0.028**	-0.023**	-0.026**
	(0.001)	(0.002)	(0.002)	(0.002)	(0.002)
Japanese * Mom Any College	-0.179**	-0.147**	-0.144**	-0.145**	-0.143**
	(0.001)	(0.001)	(0.001)	(0.001)	(0.001)
Filipino * Mom Any College	-0.030**	-0.038**	-0.037**	-0.037**	-0.036**
	(0.001)	(0.001)	(0.002)	(0.001)	(0.001)
Vietnamese * Mom Any College	-0.027**	-0.020**	-0.023**	-0.018**	-0.022**
	(0.001)	(0.001)	(0.001)	(0.001)	(0.001)
Constant	0.502**	0.521**	0.523**	0.528**	0.524**
	(0.007)	(0.021)	(0.020)	(0.025)	(0.025)
Observations	83,061	71,649	71,649	71,604	71,604
R-squared	0.000	0.001	0.001	0.001	0.001
Race/Ethnic, Educ, & Year Indicators	Y	Y	Y	Y	Y
Controls for Parental Characteristics		Y	Y	Y	Y
Controls for Cultural Measures			Y		Y
Controls for Economic Measures				Y	Y

ACS 2000-2018, limited to third children with two older sisters in households where: one parent is the household head; all children were born in the U.S.; no children are adopted or step-children; the eldest child is less than 13 years old; and children are not from multiple births.

All models include indicators for each race/ethnic category, maternal education category, and year. Full model includes controls for parental characteristics :maternal and paternal age, parental marital status; economic characteristics: separate indicators for whether the mother or father is not in the labor force, indicators for home ownership and residence on a farm, home value (logged), and family and household income (logged); and cultural measures: indicator for whether mother is not in labor force, separate indicators for whether the mother or father is foreign born or a non-citizen, separate measures for years the mother and father have resided in the U.S., indicator for living in a three-generation household (e.g., grandparents, parents, and children).

Robust standard errors adjusted for race/ethnic clustering in parentheses. ** p<0.01, * p<0.05, + p<0.1

Table B6: Predicted Likelihood of Male Third Child by Race/Ethnicity and Parental Education

	(1)	(2)	(3)	(4)
	Infant Male:Female Ratio			
VARIABLES	Dad < College		Dad ≥ College	
African American	-0.026**	-0.036**	-0.007**	0.001
	(0.000)	(0.001)	(0.000)	(0.003)
American Indian	0.025**	0.020**	0.018**	0.016**
	(0.000)	(0.001)	(0.001)	(0.003)
Chinese	0.014**	0.002	0.110**	0.134**
	(0.001)	(0.005)	(0.001)	(0.004)
Asian Indian	-0.074**	-0.083**	0.198**	0.228**
	(0.001)	(0.004)	(0.002)	(0.005)
Korean	0.061**	0.059**	0.024**	0.042**
	(0.003)	(0.003)	(0.001)	(0.003)
Japanese	0.094**	0.096**	0.202**	0.209**
	(0.003)	(0.005)	(0.002)	(0.003)
Filipino	0.012**	0.013**	0.066**	0.079**
	(0.001)	(0.003)	(0.000)	(0.003)
Vietnamese	0.057**	0.048**	-0.052**	-0.034**
	(0.001)	(0.006)	(0.001)	(0.005)
Mother Has Any College Education	0.012*	0.008+	-0.009	-0.019*
	(0.005)	(0.004)	(0.007)	(0.006)
African American * Mom Any College	0.038**	0.042**	0.017**	0.016**
	(0.000)	(0.001)	(0.000)	(0.002)
American Indian * Mom Any College	-0.062**	-0.060**	-0.046**	-0.043**
	(0.001)	(0.002)	(0.001)	(0.002)
Chinese * Mom Any College	0.100**	0.113**	-0.083**	-0.093**
	(0.001)	(0.002)	(0.001)	(0.002)
Asian Indian * Mom Any College	0.132**	0.136**	-0.113**	-0.123**
	(0.002)	(0.003)	(0.001)	(0.001)
Korean * Mom Any College	0.006	0.013*	-0.013**	-0.027**
	(0.006)	(0.004)	(0.001)	(0.001)
Japanese * Mom Any College	-0.260**	-0.259**	-0.152**	-0.152**
	(0.001)	(0.003)	(0.002)	(0.003)
Filipino * Mom Any College	-0.086**	-0.085**	-0.042**	-0.043**
	(0.002)	(0.002)	(0.001)	(0.002)
Vietnamese * Mom Any College	-0.051**	-0.044**	0.044**	0.036**
	(0.001)	(0.002)	(0.001)	(0.003)
Constant	0.505**	0.548**	0.515**	0.486**
	(0.012)	(0.029)	(0.012)	(0.019)
Observations	28,120	28,098	43,529	43,506
R-squared	0.001	0.002	0.001	0.002
Race/Ethnic, Educ, & Year Indicators	Y	Y	Y	Y
Parental, Cultural, and Economic Controls		Y		Y

ACS 2000-2018, limited to third children with two older sisters in households where: one parent is the household head; all children were born in the U.S.; no children are adopted or step-children; the eldest child is less than 13

years old; and children are not from multiple births. Models 1-2 limited to fathers with no college education. Models 3-4 limited to fathers with at least some college education.

Shaded cells indicate significant difference between coefficients by paternal education (Models 1 vs 3 and 2 vs 4).

All models include indicators for each race/ethnic category, maternal education category, and year. Full model includes controls for parental characteristics :maternal and paternal age, parental marital status; economic characteristics: separate indicators for whether the mother or father is not in the labor force, indicators for home ownership and residence on a farm, home value (logged), and family and household income (logged); and cultural measures: indicator for whether mother is not in labor force, separate indicators for whether the mother or father is foreign born or a non-citizen, separate measures for years the mother and father have resided in the U.S., indicator for living in a three-generation household (e.g., grandparents, parents, and children).

Robust standard errors adjusted for race/ethnic clustering in parentheses. ** p<0.01, * p<0.05, + p<0.1

Figure B1: Predicted Likelihood of Male Third Child by Sibling Sex and Maternal Race/Ethnicity

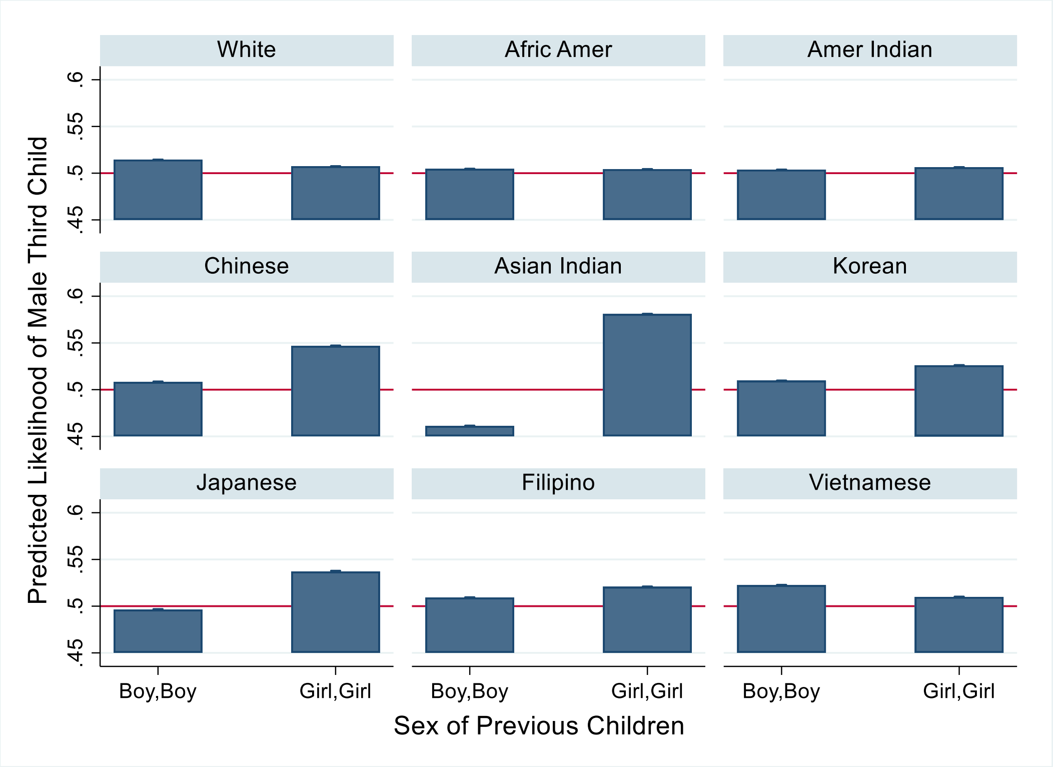

Estimates from Table B2, Model 5.

Figure B2: Predicted Age Gap between Second and Third Child with Two Previous Girls

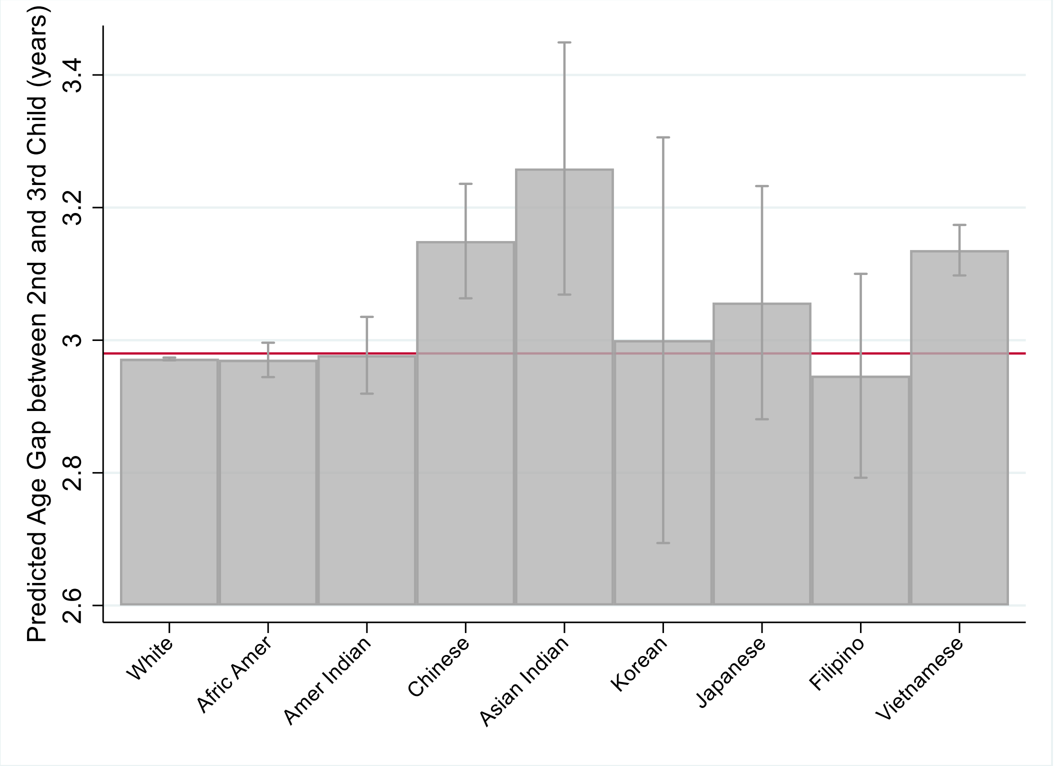

Estimates from Table B3, Model 5.
